# Impaired PPAR**γ** activation by cadmium exacerbates infection-induced lung injury

**DOI:** 10.1172/jci.insight.166608

**Published:** 2023-05-08

**Authors:** Jennifer L. Larson-Casey, Shanrun Liu, Jennifer M. Pyles, Suzanne E. Lapi, Komal Saleem, Veena B. Antony, Manuel Lora Gonzalez, David K. Crossman, A. Brent Carter

**Affiliations:** 1 Department of Medicine, Division of Pulmonary, Allergy, and Critical Care Medicine,; 2 Division of Clinical Immunology and Rheumatology, Department of Medicine,; 3 Department of Radiology,; 4 Department of Pathology, and; 5 Department of Genetics, University of Alabama at Birmingham, Birmingham, Alabama, USA.; 6 Birmingham Veterans Administration Medical Center, Birmingham, Alabama, USA.

**Keywords:** Infectious disease, Pulmonology, Bacterial infections, Innate immunity, Macrophages

## Abstract

Emerging data indicate an association between environmental heavy metal exposure and lung disease, including lower respiratory tract infections (LRTIs). Here, we show by single-cell RNA sequencing an increase in *Pparg* gene expression in lung macrophages from mice exposed to cadmium and/or infected with *Streptococcus* *pneumoniae*. However, the heavy metal cadmium or infection mediated an inhibitory posttranslational modification of peroxisome proliferator-activated receptor γ (PPARγ) to exacerbate LRTIs. Cadmium and infection increased ERK activation to regulate PPARγ degradation in monocyte-derived macrophages. Mice harboring a conditional deletion of *Pparg* in monocyte-derived macrophages had more severe *S*. *pneumoniae* infection after cadmium exposure, showed greater lung injury, and had increased mortality. Inhibition of ERK activation with BVD-523 protected mice from lung injury after cadmium exposure or infection. Moreover, individuals residing in areas of high air cadmium levels had increased cadmium concentration in their bronchoalveolar lavage (BAL) fluid, increased barrier dysfunction, and showed PPARγ inhibition that was mediated, at least in part, by ERK activation in isolated BAL cells. These observations suggest that impaired activation of PPARγ in monocyte-derived macrophages exacerbates lung injury and the severity of LRTIs.

## Graphical abstract

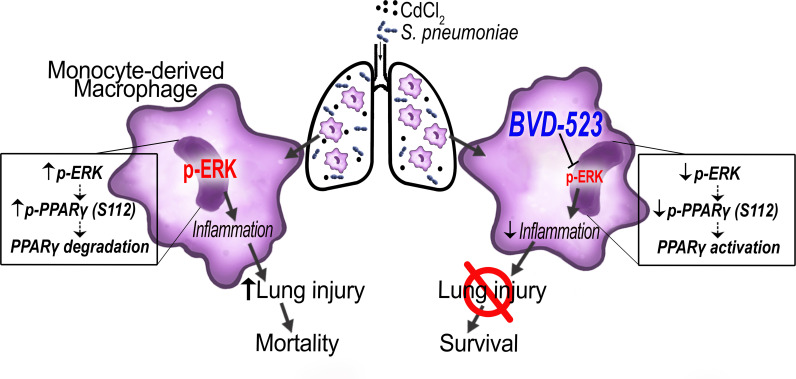

## Introduction

Lower respiratory tract infections (LRTIs), including bacterial pneumonia, are a leading cause of adult morbidity and mortality in the United States and ranked fourth as the leading cause of death worldwide (1, 2). Approximately 120–156 million cases of LRTIs are reported annually worldwide, leading to 1.4 million deaths (3). The primary infectious cause of respiratory failure is bacterial LRTI; in particular, *Streptococcus pneumoniae* is the leading cause of morbidity and mortality associated with LRTIs, accounting for over 55% of LRTI deaths (3).

The adverse effects of air pollution are linked to many diseases, including cardiopulmonary disease, stroke, lung cancer, chronic respiratory disorders, and respiratory infections (4–6), and leads to substantial economic and healthcare costs (4). The component of air pollution containing particulate matter smaller than 2.5 μm in diameter (PM_2.5_) has been identified as a potential contributor to respiratory infection in adults and children (7–9). Short-term increases in PM_2.5_ are also associated with increased rates of hospitalization secondary to respiratory infections (9, 10). Cadmium is enriched in PM_2.5_ and has the capability to reach the alveolar space, which is particularly relevant in LRTIs (11). Widely distributed in the environment, natural air emission sources of cadmium can come from volcanoes, airborne soil particles, forest fires, coal-fired plants, coke factories, and quarries (11). Cadmium adversely affects lung function and contributes to pulmonary fibrosis, cancer, asthma, and chronic obstructive pulmonary disease (12–14). Although environmental cadmium was recently identified to be associated with a higher risk of mortality from influenza or pneumonia (15), the mechanism(s) regulating cadmium-mediated respiratory infections is not known.

Lung macrophages play a critical role in host defense against respiratory pathogens (16). Bone marrow–derived monocytes are recruited to the lung during infection in a C-C chemokine receptor type 2–dependent (CCR2-dependent) manner (17). The increase in macrophage number seen in *S*. *pneumonia*–infected mice is due to macrophage recruitment, rather than expansion of the tissue-resident alveolar macrophage (TRAM) population (18). Recruitment of monocyte-derived macrophages (MDMs) worsened *S*. *pneumoniae* infection in mice previously infected with influenza (19), which may be attributed to impaired efferocytosis and clearance of *S*. *pneumoniae* (20). Moreover, MDMs contributed to enhanced inflammatory response and disease progression in several models of lung disease (21–26).

Our previous work showed that lung macrophages play a critical role in host defense against respiratory pathogens. Macrophage depletion significantly increased bacterial lung burden in mice, while no difference was detected in mice depleted of neutrophils (21). A recent study showed that cadmium-mediated lung injury resulted in the persistence of classically activated lung macrophages by inhibiting the nuclear localization of peroxisome proliferator-activated receptor γ (PPARγ) (27). PPARγ, a ligand-activated transcription factor belonging to the nuclear receptor superfamily, is a negative regulator of the inflammatory response (28). PPARγ is known to inhibit production of proinflammatory cytokines and reactive oxygen species generation. PPARγ also controls the alternative activation of monocytes and macrophages (29).

PPARγ contains a consensus mitogen-activated protein kinase (MAPK) site, and phosphorylation at serine 112 inhibits PPARγ activation via degradation (30). The extracellular signal–regulated kinase (ERK) plays a pivotal role in lung inflammation (31) and has been suggested to be increased in mouse models of lung injury; however, the mechanism by which it contributes to lung injury has not been determined. We hypothesized that the heavy metal cadmium regulates an inhibitory posttranslational modification of PPARγ to exacerbate LRTIs. Here, we show that recruited lung macrophages have a critical role in mounting an immune response to foreign agents to promote host defense, but also contribute to the pathogenesis of lung injury by impairment of PPARγ activation via the activation of ERK. These observations suggest that PPARγ is critical in regulating lung injury during LRTIs.

## Results

### Infection is exacerbated in mice exposed to cadmium.

**Figure 1:**
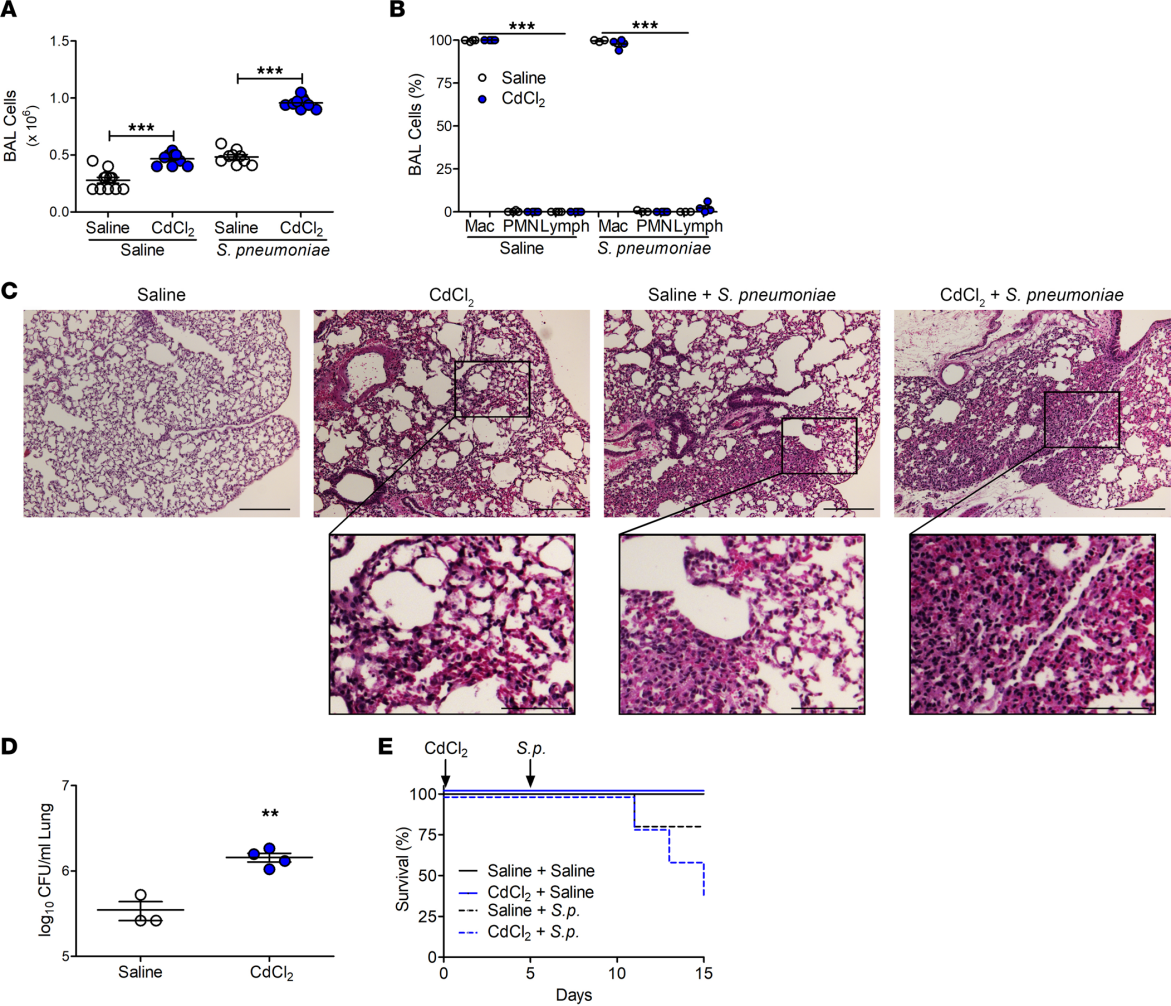
Cadmium exacerbates lower respiratory tract infections. WT mice were exposed to saline or CdCl_2_ (100 ng/kg) by i.t. administration. On day 5, mice were exposed to saline or 1 × 10^3^ *Streptococcus pneumoniae* (strain A66.1, type 3) i.t. and BAL was performed on day 15. (**A**) Number of BAL cells. *n* = 9–17. (**B**) Cell differential to identify macrophages (Mac), neutrophil (PMN), and lymphocytes (Lymph). *n* = 3–5. (**C**) Representative hematoxylin and eosin staining of lung tissues. *n* = 3–5. Scale bars: 250 μm and 100 μm (insets). (**D**) Lung CFUs. *n* = 3–4. (**E**) Kaplan-Meier survival curves. *n* = 4–5. Data shown as mean ± SEM. **P < 0.001; ***P < 0.0001 by 1-way ANOVA with Tukey’s post hoc test (**A** and **B**) or 2-tailed Student’s *t* test (**D**).

Because cadmium is associated with increased risk of mortality from pneumonia (15), we investigated whether cadmium altered the innate immune response to bacterial infections. Cadmium-exposed mice had increased numbers of bronchoalveolar lavage (BAL) cells that increased further after exposure to *S*. *pneumoniae* (strain A66.1, type 3), as a model of LRTI (Figure 1A). Macrophages were the predominant cell type in the BAL fluid (BALF) from cadmium-exposed and *S*. *pneumoniae*–infected mice throughout the duration after exposure. Neutrophils, however, only showed a transient increase after cadmium exposure and *S*. *pneumoniae* infection (Figure 1B and Supplemental Figure 1, A–C; supplemental material available online with this article; https://doi.org/10.1172/jci.insight.166608DS1). The lungs of cadmium-exposed mice had thickened alveolar septa and cellular inflammation, and *S*. *pneumoniae*–infected mice had similar findings as well as areas of consolidation (Figure 1C). The combination of cadmium and *S*. *pneumoniae* infection led to significantly greater consolidation with multilobar involvement in mice. The histological findings were confirmed, as cadmium-exposed mice had significantly greater lung colony-forming units (CFUs) than saline-exposed mice (Figure 1D). Moreover, cadmium-exposed mice had significantly greater mortality after infection (Figure 1E). These data indicate that cadmium exposure increases the severity of LRTI.

### PPARγ is primarily expressed in macrophages.

**Figure 2:**
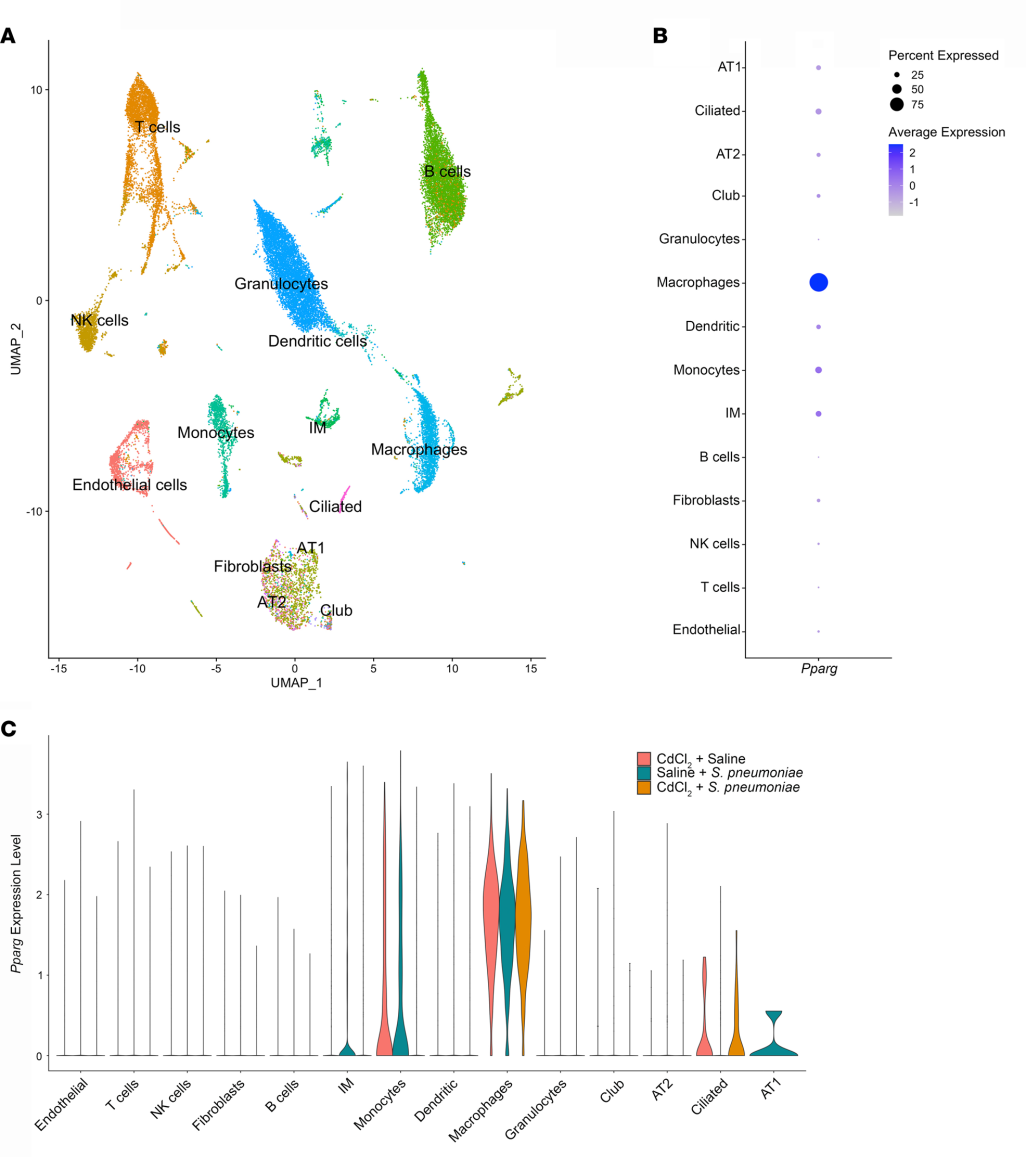
Single-cell RNA sequencing identifies *Pparg* expression in macrophages. WT mice were exposed to saline or CdCl_2_ by i.t. administration. On day 5, mice were exposed to saline or *Streptococcus* *pneumoniae* i.t. and lungs were harvested on day 15. (**A**) Uniform manifold approximation and projection (UMAP) plot identifying 15 cell clusters by single-cell RNA sequencing (1 mouse per condition). (**B**) Dot plot of *Pparg* expression in all cell clusters. (**C**) Violin plot of *Pparg* expression in exposed mice in each cell cluster. AT1 and AT2, alveolar epithelial type I and II cells; IM, interstitial macrophages.

To understand the cellular populations and mediators that increase the severity of LRTI, we performed single-cell RNA sequencing on unenriched single-cell suspensions from lung tissue of exposed mice. After quality filtering, we obtained 29,204 cell profiles from all samples. Analysis of representative markers identified all major cell types within the mouse lung (Figure 2A).

Because PPARγ plays a critical role in macrophage differentiation, we determined that *Pparg* was primarily expressed in macrophages (Figure 2B and Supplemental Figure 2A). Compared with all identified clusters, the robust expression of *Pparg* was maintained in macrophages in all exposure conditions (Figure 2C and Supplemental Figure 2B). Compared with the other identified isoforms of *Ppar*, *Pparg* showed the greatest expression in the macrophage cluster (Supplemental Figure 2C).

### Cadmium mediated PPARγ phosphorylation at Ser^112^, resulting in greater lung injury.

**Figure 3:**
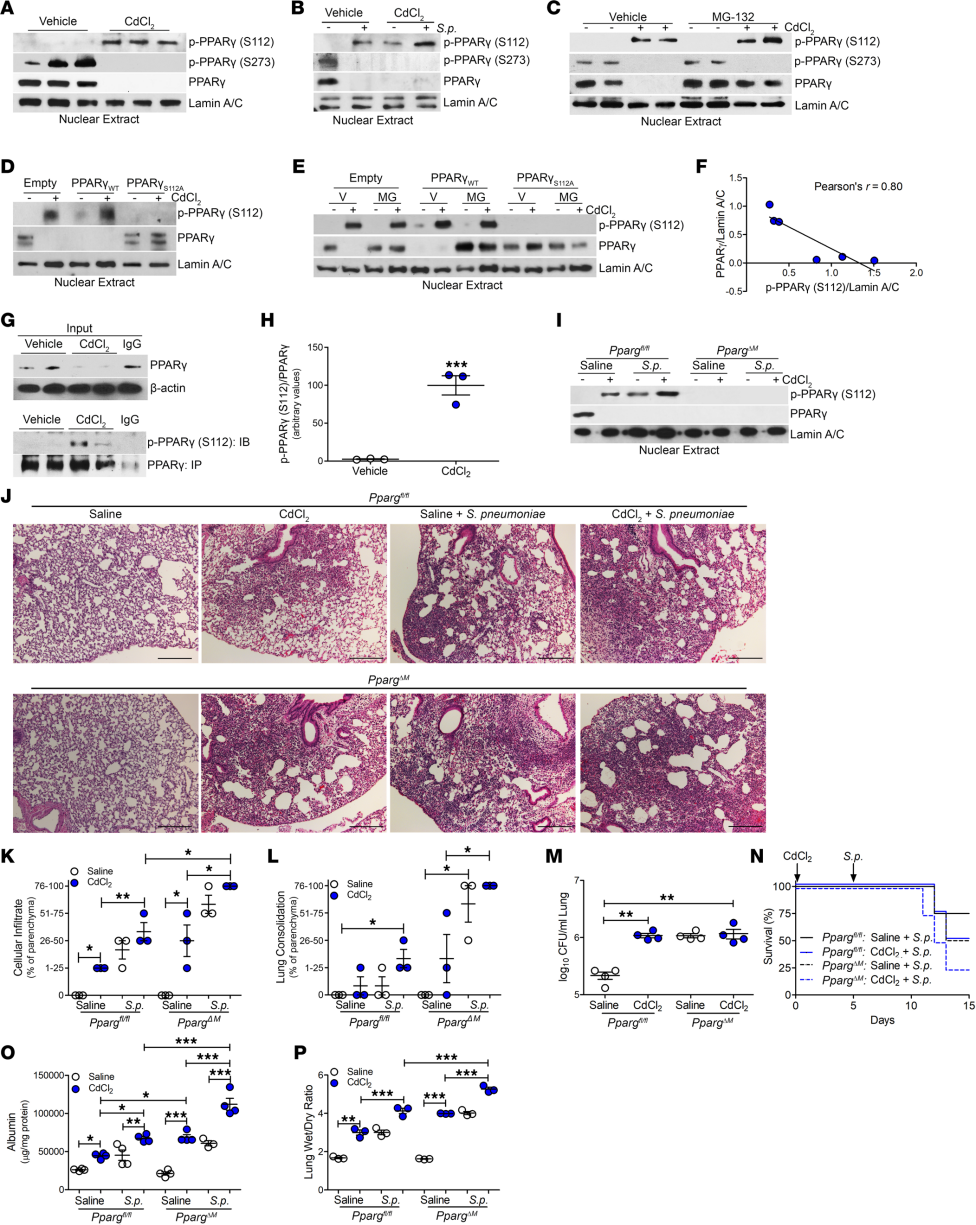
Cadmium mediated PPARγ phosphorylation at Ser^112^, resulting in greater lung injury. (**A**) Nuclear immunoblot analysis of THP-1 cells exposed to CdCl_2_ (50 μM, 3 hours). (**B**) Nuclear immunoblot analysis of BAL cells from exposed WT mice. (**C**) Immunoblot analysis of isolated nuclear extracts of THP-1 cells treated with vehicle or MG-132 (20 μM, 6 hours) and saline or CdCl_2_ (50 μM, 3 hours). (**D**) Immunoblot analysis of isolated nuclear extracts of THP-1 cells expressing empty vector, PPARγ_WT_, or PPARγ_S112A_ treated with saline or CdCl_2_. (**E**) Immunoblot analysis of isolated nuclear extracts of THP-1 cells expressing empty vector, PPARγ_WT_, or PPARγ_S112A_ treated with vehicle or MG-132. (**F**) Pearson’s correlation of densitometry of phosphorylated PPARγ (S112) and PPARγ relative to Lamin A/C in transfected THP-1 cells treated with vehicle in **E**. (**G**) Immunoprecipitation of PPARγ from cadmium-exposed THP-1 cells with (**H**) statistical quantification of phosphorylated PPARγ (S112) relative to PPARγ in **G**. (**I**) Nuclear immunoblot analysis of BAL cells from exposed *Pparg^fl/fl^* and *Pparg*^ΔM^ mice. (**J**) Representative hematoxylin and eosin staining of lung tissues. *n* = 3–5. Scale bars: 250 μm. (**K** and **L**) Scoring of lung tissue from **J** for (**K**) cellular infiltrate and (**L**) consolidation. (**M**) Lung CFUs. *n* = 4. (**N**) Kaplan-Meier survival curves. *n* = 4–5. (**O**) Albumin levels in BALF. *n* = 3–4. (**P**) Wet to dry ratio of lung weight from exposed mice. *n* = 3. Data shown as mean ± SEM. *P < 0.05; **P < 0.001; ***P < 0.0001 by 1-way ANOVA with Tukey’s post hoc test. Pearson’s coefficient was used for **F**.

We investigated whether cadmium regulated PPARγ by altering the posttranslational modification of PPARγ. Cadmium exposure led to a marked increase in PPARγ phosphorylation at its Ser^112^ residue, which correlated with the absence of PPARγ nuclear localization; however, cadmium exposure did not lead to the phosphorylation of PPARγ at its Ser^273^ residue (Figure 3A and Supplemental Figure 3, A–C). Similar results were obtained in BAL cells isolated from infected mice. Cadmium exposure mediated the phosphorylation of PPARγ at Ser^112^ to a level similar to that seen in *S*. *pneumoniae*–infected mice (Figure 3B and Supplemental Figure 3D). Cadmium-exposed and *S*. *pneumoniae*–infected mice showed enhanced phosphorylation of PPARγ at Ser^112^ and an absence of phosphorylation of PPARγ at Ser^273^ and nuclear PPARγ expression (Supplemental Figure 3, E and F). Because phosphorylation can influence PPARγ degradation (32), phosphorylation at Ser^112^ was linked to PPARγ degradation, as treatment with the proteasome inhibitor MG-132 resulted in PPARγ accumulation in the nucleus of cadmium-exposed macrophages (Figure 3C and Supplemental Figure 3, G–I).

Validating that cadmium regulates the phosphorylation of PPARγ at Ser^112^, we mutated Ser^112^ to alanine (PPARγ_S112A_). Absence of PPARγ (S112) phosphorylation in macrophages expressing the S112A mutant resulted in nuclear localization of PPARγ in cadmium-exposed macrophages, whereas macrophages expressing PPARγ_WT_ showed increased p-PPARγ (S112) expression and absent PPARγ nuclear localization (Figure 3D and Supplemental Figure 3, J and K). Confirming that cadmium mediated the degradation of PPARγ via phosphorylation at Ser^112^, cadmium-exposed macrophages expressing PPARγ_WT_ had increased p-PPARγ (S112) expression; however, inhibiting degradation in the proteosome resulted in PPARγ nuclear expression (Figure 3E and Supplemental Figure 3, L and M). Macrophages expressing the mutant plasmid (S112A) maintained PPARγ nuclear localization regardless of treatment with MG-132. The absence of nuclear PPARγ expression was inversely correlated with p-PPARγ (S112) expression in macrophages treated with cadmium (Figure 3F). Quantitatively determining the fraction of PPARγ phosphorylated at Ser^112^ after cadmium exposure, lysates were subjected to PPARγ immunoprecipitation. Cadmium-exposed macrophages showed increased p-PPARγ (S112) expression, with an absence in vehicle exposed, indicating nearly all of PPARγ is phosphorylated at Ser^112^ after cadmium exposure (Figure 3, G and H). Similar results were obtained in PPARγ_WT_-His–transfected macrophages treated with cadmium (Supplemental Figure 3N).

To determine the physiologic role of cadmium and infection in the regulation in the posttranslational modification of PPARγ, we generated mice harboring a conditional deletion of *Pparg* in macrophages (*Pparg*^ΔM^). Cadmium-exposed or *S*. *pneumoniae*–infected *Pparg^fl/fl^* mice showed p-PPARγ (S112) expression in the nuclear fraction of isolated lung macrophages (Figure 3I). Phosphorylation of PPARγ at Ser^112^ was further increased in cadmium-exposed *Pparg^fl/fl^* mice after *S*. *pneumoniae* infection. *Pparg*^ΔM^ mice showed an absence of PPARγ regardless of exposure. The conditional deletion of *Pparg* did not alter normal lung architecture; however, *Pparg*^ΔM^ mice exposed to cadmium or infected with *S*. *pneumoniae* had greater cellular inflammation and consolidation. Essentially all the lung was consolidated in *Pparg*^ΔM^ mice exposed to cadmium followed by infection, whereas the *Pparg^fl/fl^* mice had partial consolidation (Figure 3J). The degree of lung inflammation and consolidation was quantified by scoring lung sections from both strains (Figure 3, K and L). Interestingly, there was no difference in CFUs in the lung between these strains of mice (Figure 3M); however, infected *Pparg*^ΔM^ mice had markedly reduced survival and a reduction in body weight (Figure 3N and Supplemental Figure 3, O and P). Rather than increased bacterial burden, the increased mortality was associated with greater loss of barrier function and increased ratio of wet to dry lung weight, indicating more severe lung injury in the *Pparg*^ΔM^ mice (Figure 3, O and P). These observations suggest that the severity of LRTI was secondary to increased lung injury via the impaired activation of PPARγ.

### ERK activation mediates phosphorylation of PPARγ.

**Figure 4:**
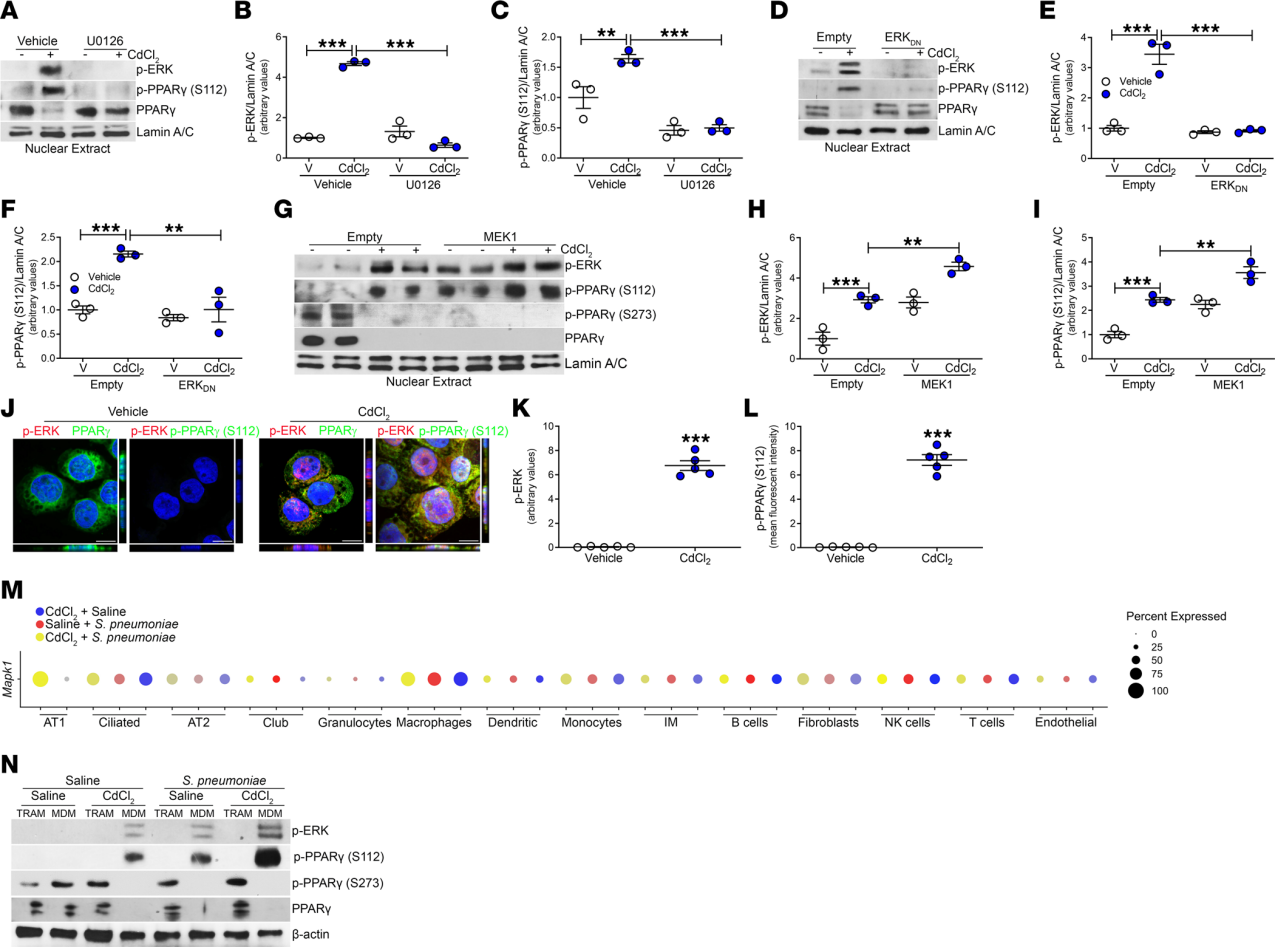
ERK activation mediates phosphorylation of PPARγ. (**A**) Nuclear immunoblot analysis of THP-1 cells exposed to vehicle or U0126 (10 μM, 1 hour) and CdCl_2_ (50 μM, 3 hours) with statistical quantification of (**B**) p-ERK and (**C**) p-PPARγ (S112). *n* = 3. (**D**) Nuclear immunoblot analysis of THP-1 cells transfected with empty vector or ERK_DN_ and exposed to saline or CdCl_2_, with statistical quantification of (**E**) p-ERK and (**F**) p-PPARγ (S112). *n* = 3. (**G**) Nuclear immunoblot analysis of THP-1 cells transfected with empty vector or MEK1 and exposed to saline or CdCl_2_, with statistical quantification of (**H**) p-ERK and (**I**) p-PPARγ (S112) *n* = 3. (**J**) Representative confocal imaging of exposed MH-S cells. Scale bars: 10 μm. Statistical quantification of (**K**) p-ERK and (**L**) p-PPARγ (S112) staining *n* = 5. (**M**) Dot plot of percentage of *Mapk1* expression in each cell cluster in exposed mice from lung tissue analyzed by single-cell RNA sequencing. AT1 and AT2, alveolar epithelial type I and II cells; IM, interstitial macrophages. (**N**) Immunoblot analysis of FACS-isolated BAL cells from exposed WT mice. Tissue-resident alveolar macrophages (TRAMs; CD45^+^CD11b^+/–^Ly6G^–^CD64^+^Ly6C^–^Siglec F^hi^) and monocyte-derived macrophages (MDMs; CD45^+^CD11b^+/–^Ly6G^–^CD64^+^Ly6C^–^Siglec F^lo^). Data shown as mean ± SEM. **P < 0.001; ***P < 0.0001 by 1-way ANOVA with Tukey’s post hoc test (**B**, **C**, **E**, **F**, **H**, and **I**) or 2-tailed Student’s *t* test (**K** and **L**).

Mitogen-activated protein kinases (MAPKs) regulate activation of multiple transcription factors (33), including the suppression of PPARγ transcriptional activity by phosphorylation at Ser^112^ (34). Because cadmium was previously shown to augment NF-κB activity that resulted in the persistence of a proinflammatory phenotype in lung macrophages (27), we investigated whether the extracellular signal–related kinase (ERK) was activated, as ERK is an essential regulator of NF-κB activity (33, 35). While cadmium increased ERK activity, cadmium-mediated ERK activation was inhibited in macrophages treated with a MEK inhibitor, U0126 (Figure 4, A and B), or macrophages expressing a dominant negative ERK (ERK_DN_) (Figure 4, D and E). ERK inhibition induced PPARγ nuclear localization and inhibited phosphorylation of PPARγ (S112) (Figure 4, C and F, and Supplemental Figure 4, A and B). Macrophages expressing constitutively active MEK1 showed ERK activation that increased further after cadmium exposure (Figure 4, G and H). Constitutively active MEK1 alone or when combined with cadmium exposure led to PPARγ inhibition with increased p-PPARγ (S112) expression, whereas phosphorylation at the Ser^273^ residue was not altered (Figure 4I and Supplemental Figure 4, C and D). These findings were specific to cadmium, as other metals in particulate matter, such as arsenic or manganese, did not inhibit PPARγ nuclear localization or alter phosphorylation at Ser^112^ via ERK activation (Supplemental Figure 4E). Visually confirming these results, vehicle-exposed macrophages had an absence of ERK activation, nuclear localization of PPARγ, and absence of PPARγ (S112) phosphorylation, whereas cadmium mediated ERK activation and p-PPARγ (S112) expression that caused retention of PPARγ in the cytoplasm (Figure 4, J–L, and Supplemental Figure 4F).

To further investigate regulation of PPARγ, single-cell RNA sequencing in lung tissue from exposed mice showed high expression of *Mapk1* in the macrophage cell cluster under all exposure conditions (Figure 4M and Supplemental Figure 3, G and H). Validating these results, FACS-isolated BAL cells were analyzed by immunoblotting. Cadmium exposure and *S*. *pneumoniae* infection mediated ERK activation and phosphorylation of PPARγ (S112) only in MDMs (Figure 4N and Supplemental Figure 4I). PPARγ nuclear expression was detected in the TRAMs and MDMs in saline-exposed mice. MDMs from exposed or infected mice showed an absence of PPARγ nuclear localization, whereas nuclear expression of PPARγ was retained in TRAMs. To determine whether PPARγ was functionally active in TRAMs, we measured gene expression of arginase 1 (*Arg1*), as PPARγ binds to its promoter to induce transcription (29). PPARγ-dependent *Arg1* gene expression was minimally expressed in TRAMs from cadmium-exposed mice infected with *S*. *pneumoniae* and remained at the same level regardless of the exposure condition (Supplemental Figure 4J). Taken together, these data show that MDM activation was regulated by ERK-mediated phosphorylation of PPARγ at Ser^112^.

### Severe LRTIs inhibit PPARγ expression in MDMs.

**Figure 5:**
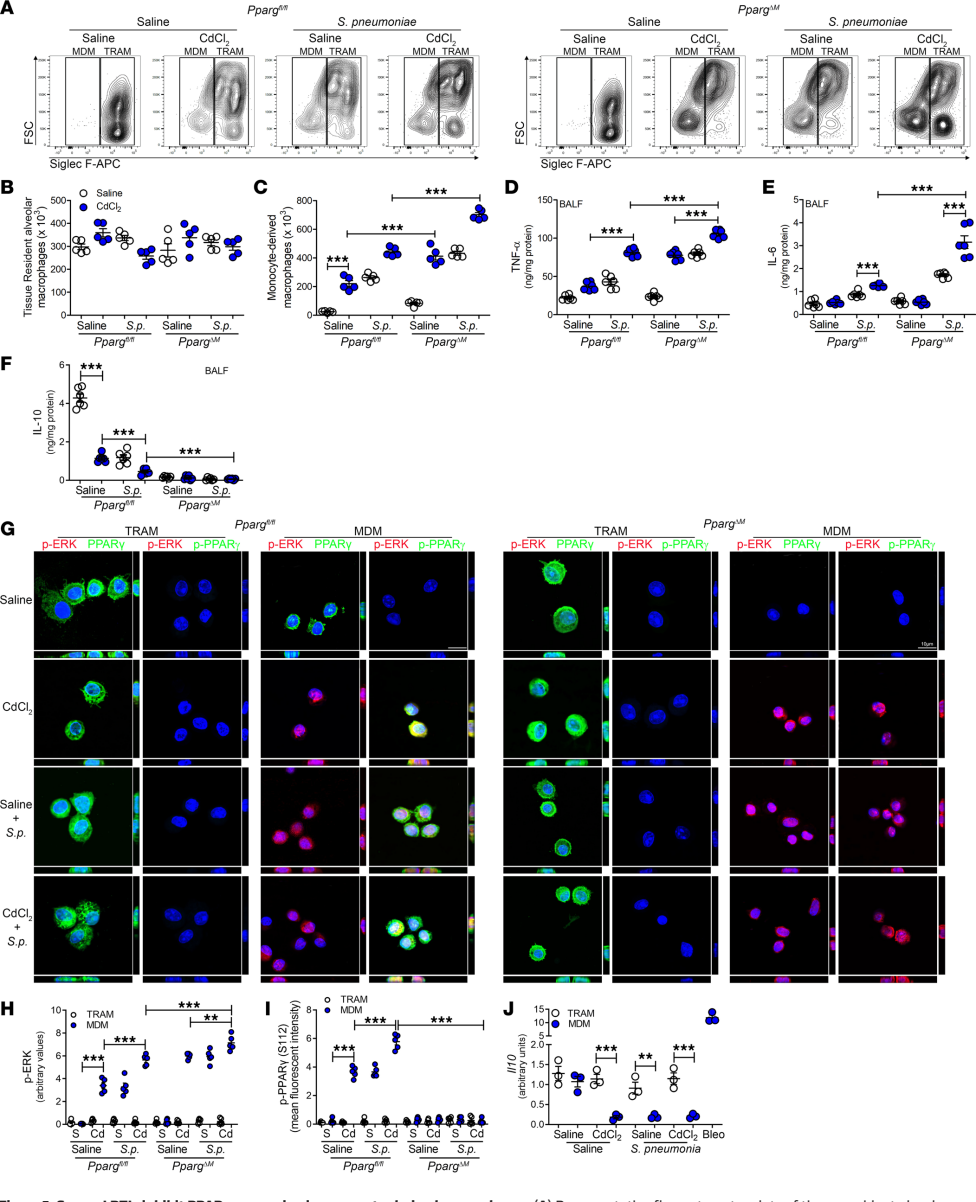
Severe LRTIs inhibit PPARγ expression in monocyte-derived macrophages. (**A**) Representative flow cytometry plots of tissue-resident alveolar macrophages (TRAMs; CD45^+^CD11b^+/–^Ly6G^–^CD64^+^Ly6c^–^Siglec F^hi^) and monocyte-derived macrophages (MDMs; CD45^+^CD11b^+/–^Ly6G^–^CD64^+^Ly6c^–^Siglec F^lo^) from exposed *Pparg^fl/fl^* and *Pparg^–/–^* *Lyz2-cre* mice on day 15. Number of (**B**) TRAMs and (**C**) MDMs in BALF. *n* = 5. (**D**) TNF-α, (**E**) IL-6, and (**F**) IL-10 levels in BALF. *n* = 6. (**G**) Representative confocal imaging of FACS-isolated BAL cells. *n* = 3. Scale bars: 10 μm. Statistical quantification of confocal imaging of (**H**) p-ERK and (**I**) p-PPARγ (S112) staining. *n* = 5. (**J**) *Il10* mRNA expression in FACS-isolated BAL cells. *n* = 3. Data shown as mean ± SEM. **P < 0.001; ***P < 0.0001 by 1-way ANOVA with Tukey’s post hoc test.

Because our data suggest that cadmium regulates PPARγ expression in MDMs and is associated with more severe LRTIs, we further investigated the role of macrophage recruitment to the lung. Cadmium exposure and *S*. *pneumoniae* infection significantly increased MDMs in *Pparg^fl/fl^* and *Pparg*^ΔM^ mice (Figure 5, A and C). *Pparg*^ΔM^ mice infected with *S*. *pneumoniae* after cadmium exposure showed significantly more MDMs than were seen in *Pparg^fl/fl^* mice. In contrast, the number of TRAMs remained unchanged between strains and exposures (Figure 5, A and B). The recruitment of MDMs correlated with increased TNF-α levels in the BALF, and *Pparg*^ΔM^ mice showed greater levels after cadmium and infection than *Pparg^fl/fl^* mice (Figure 5D). IL-6, which has been associated with greater mortality, was increased in infected mice, and was markedly greater in *Pparg*^ΔM^ mice previously exposed to cadmium (Figure 5E). Because PPARγ has been shown to regulate IL-10 production during bacterial infection (36, 37), we found that cadmium exposure and *S*. *pneumoniae* infection reduced IL-10 levels in the *Ppar^fl/fl^* mice, and the *Pparg*^ΔM^ mice showed an even greater reduction (Figure 5F). Validating that MDMs are responsible for the inflammatory response to cadmium exposure, *Ccr2^–/–^* mice showed a significant reduction in the inflammatory mediators, TNF-α and inducible NOS, compared with cadmium-exposed WT mice (Supplemental Figure 5, A–D).

To further confirm the role of PPARγ in MDMs, FACS-isolated BAL cells were analyzed by confocal microscopy. PPARγ was expressed in TRAMs from both strains regardless of exposure (Figure 5G and Supplemental Figure 5E). MDMs from *Pparg^fl/fl^* mice showed ERK activation and PPARγ (S112) phosphorylation after cadmium exposure or infection (Figure 5, H and I). Although MDMs from *Pparg*^ΔM^ mice did not express PPARγ or p-PPARγ (S112), cadmium and *S*. *pneumoniae* infection induced ERK activation. Moreover, the absence of PPARγ expression in MDMs from exposed mice was associated with markedly reduced IL-10 expression, whereas TRAMs showed no change in IL-10 (Figure 5J). These data suggest that the exacerbated lung injury in LRTIs is, in part, due to ERK activation in MDMs.

### Individuals residing in areas with high air cadmium levels show PPARγ inhibition.

**Figure 6:**
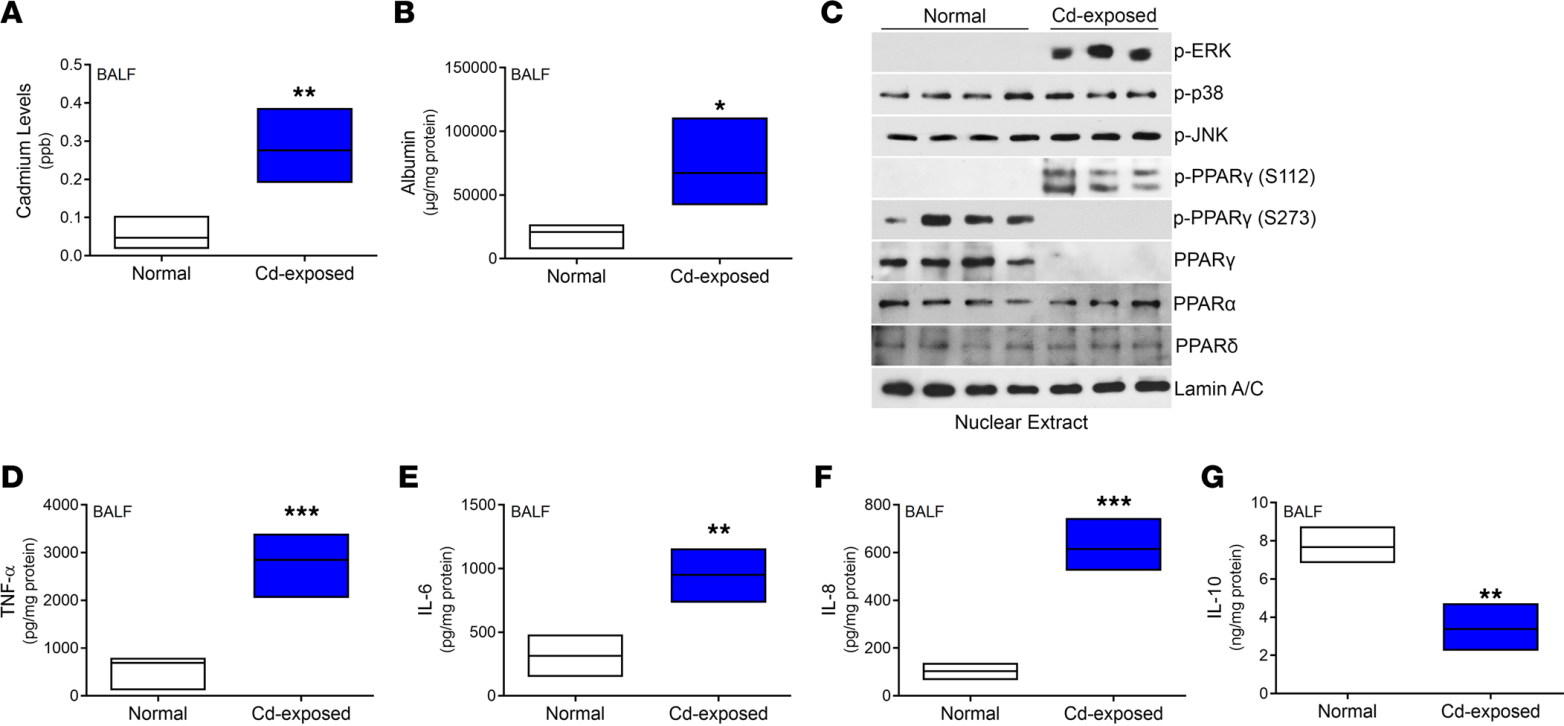
Individuals residing in areas with high air cadmium levels show PPARγ inhibition. (**A**) BALF samples from control and cadmium-exposed individuals were analyzed by inductively coupled plasma mass spectrometry to determine cadmium levels. *n* = 3–4. (**B**) Albumin levels in BALF from control and cadmium-exposed individuals. *n* = 3–4. (**C**) Nuclear immunoblot of BAL cells from normal and cadmium-exposed individuals. Levels of (**D**) TNF-α, (**E**) IL-6, (**F**) IL-8, and (**G**) IL-10 in BALF. *n* = 3–4. Data shown as mean ± SEM. *P < 0.05; **P < 0.001; ***P < 0.0001 by 2-tailed Student’s *t* test.

Neighborhoods surrounding industrial complexes have increased health risks from the continuous exposure to hazardous compounds released during industrial activity. We obtained BALF from individuals residing in an area with a significant industrial legacy as well as continuously active industry, including coal-fired plants, coke factories, and quarries. Compared with individuals from a control area where industrial activity was not present, those residing near the active industry had increased levels of cadmium in their BALF (Figure 6A). The increased cadmium levels were associated with a loss of epithelial barrier function (Figure 6B). BAL cells isolated from cadmium-exposed individuals showed absence of PPARγ localization in the nucleus, while the PPARα and PPARδ isoforms were not altered (Figure 6C). Furthermore, ERK activation and phosphorylation of PPARγ at Ser^112^ was present in cadmium-exposed individuals, but it was absent in controls. Cadmium-exposed individuals did not show p38 or c-Jun N-terminal kinase (JNK) activation, and phosphorylation of PPARγ at Ser^273^ was not detected. TNF-α, IL-6, and IL-8 were significantly increased in the BALF from cadmium-exposed individuals (Figure 6, D–G); however, IL-10 levels were reduced, suggesting that the lungs of cadmium-exposed individuals were in a proinflammatory state at baseline.

### Inhibition of ERK reduces lung injury, facilitating PPARγ activation during LRTI.

**Figure 7:**
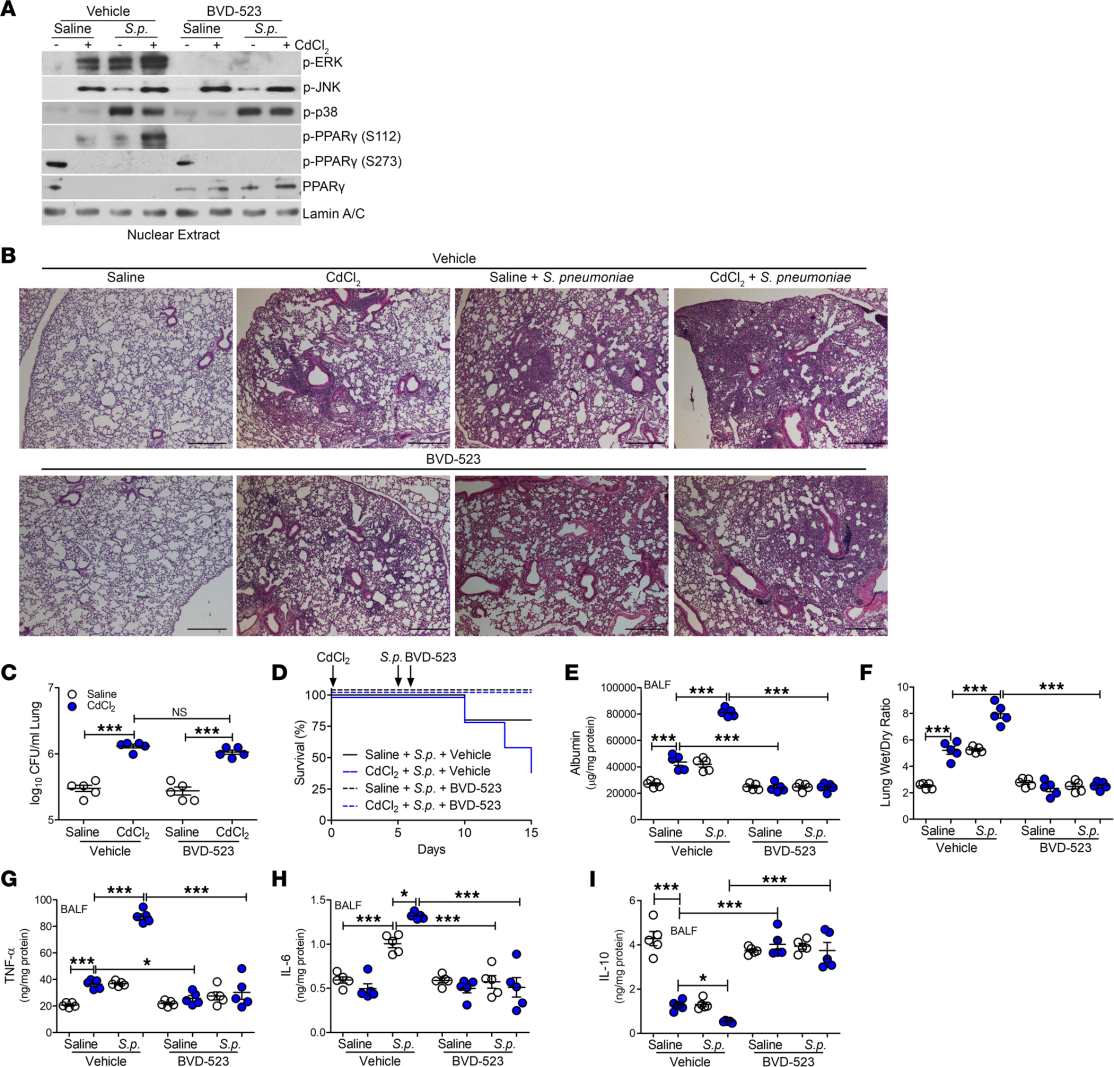
Inhibition of ERK activation reduces lung injury, facilitating PPARγ activation during LRTI. WT mice were exposed to saline or CdCl_2_ (100 ng/kg) by i.t. administration. On day 5, mice were exposed to saline or 1 × 10^3^ *Streptococcus pneumoniae* (strain A66.1, type 3) i.t. Vehicle or BVD-523 (8.6 mg/kg) was administered twice daily to mice starting on day 6 until day 15. BAL was performed on day 15. (**A**) Nuclear immunoblot analysis of BAL cells from exposed mice. (**B**) Representative hematoxylin and eosin staining of lung tissues. *n* = 5. Scale bars: 250 μm. (**C**) Lung CFUs. *n* = 5. (**D**) Kaplan-Meier survival curves. *n* = 5. (**E**) Albumin levels in BALF. *n* = 5. (**F**) Wet to dry ratio of lung weight from exposed mice. *n* = 5. (**G**) TNF-α, (**H**) IL-6, and (**I**) IL-10 levels in BALF. *n* = 5. Data shown as mean ± SEM. *P < 0.05; ***P < 0.0001 by 1-way ANOVA with Tukey’s post hoc test.

To investigate the requirement for ERK activation mediating lung injury, we used BVD-523, an inhibitor of ERK activation. BVD-523, a small-molecule ERK1/2 kinase inhibitor, is currently under investigation in phase II clinical trials for cancer therapy (38). Its effect in altering lung injury is not known. WT mice were exposed to saline or cadmium and infected with *S*. *pneumoniae*. Mice were administered vehicle or BVD-523 twice daily, starting 1 day after infection until day 15. There was no difference in the BAL cell differential, with the majority being a monocytic cell type (Supplemental Figure 6A). BVD-523 did not induce apoptosis, suggesting it was not toxic (Supplemental Figure 6B). BVD-523 inhibited ERK activation in cadmium-exposed or infected mice; however, BVD-523 did not alter p38 and JNK activation in mice (Figure 7A). PPARγ phosphorylation at Ser^112^ was absent in isolated lung macrophages from mice treated with BVD-523, and BVD-523 did not alter phosphorylation at Ser^273^. Furthermore, the treatment of mice with BVD-523 also rescued nuclear localization of PPARγ in lung macrophages regardless of exposure.

BVD-523 did not alter normal lung architecture in saline-exposed mice (Figure 7B). Mice receiving vehicle showed cellular inflammation and consolidation after exposure to cadmium or infection with *S*. *pneumoniae*. Administration of BVD-523 did not alter consolidation or CFUs in the lung (Figure 7, B and C); however, compared with mice administered vehicle, mice receiving BVD-523 showed increased survival, increased barrier function, and reduced ratio of wet to dry lung weight, suggesting a reduction in lung injury (Figure 7, D–F). The reduced lung injury in LRTI correlated with reduced TNF-α and IL-6 levels in mice treated with BVD-523 (Figure 7, G and H). Moreover, IL-10 levels were increased in mice receiving BVD-523 (Figure 7I). In aggregate, these observations uncover a molecular mechanism by which cadmium exacerbates LRTIs and lung injury. These findings further suggest that inhibiting ERK activation in MDMs to recover PPARγ activation may be a novel therapeutic modality to lessen the severity of infection and lung injury in individuals living in areas with high levels of cadmium.

## Discussion

Although lung injury after respiratory infection is often unavoidable, identifying modifiable risk factors that predispose individuals to severe pneumonia is an unmet need. Epidemiological studies have provided evidence on the adverse effects of exposure to airborne particles. Exposure to fine particulate matter (PM_2.5_) is associated with increased rates of lung function decline, mortality, and respiratory morbidity (5, 6, 39–41). Recent data indicate that PM_2.5_-associated deaths within the United States greatly affect individuals living in communities with greater socioeconomic deprivation, as well as non-Hispanic Black or African American individuals (42). Although the composition of PM_2.5_ was not determined in this study, PM_2.5_ is enriched with the heavy metal cadmium (43, 44), and exposure to cadmium has been shown to double the risk of lung disease (13). Here, we show that prior exposure to cadmium exacerbates LRTIs by enhancing the degradation of PPARγ within specific lung macrophage subsets.

MDMs contribute to lung injury and lung remodeling by increased recruitment to the lung (27, 45, 46). The persistence of these cells within the lung months after initial injury may exacerbate lung injury and contribute to lasting health consequences, in part due to the robust proinflammatory response of recruited MDMs. Evidence suggests that TRAMs and MDMs respond differently to lung injury (47). The number of MDMs in the lung may contribute to exacerbated inflammatory responses and disease severity, which was recently identified in COVID-19 patients (26).

PPARγ, a ligand-activated nuclear receptor, is a negative regulator of the inflammatory response by inhibiting production of proinflammatory cytokines, including TNF-α and IL-6 (28). PPARγ activation opposes inflammation in macrophages most notably by inhibiting inflammatory transcription factors, such as NF-κB and AP-1, and increases expression of IL-10 (29). Controversy exists regarding the role of PPARγ in bacterial infections. Conditional deletion of PPARγ in myeloid cells in mice showed a hyperinflammatory response to infections to promote pathogen clearance in response to *Mycobacterium tuberculosis*, *Salmonella typhimurium*, *Brucella abortus*, and *Listeria monocytogenes* infection (48–50).

Phosphorylation of PPARγ at Ser^112^ was recently shown to be mediated by the upstream JNK-MAPK in a murine acute lung injury model (36). Although cadmium exposure promoted JNK activation, our data demonstrate that ERK activation was, at least in part, responsible for the increased lung injury seen in these mice, and ERK inhibition did not alter JNK activation. ERK has also been shown to phosphorylate PPARγ at Ser^273^ and Ser^133^ (51). Phosphorylation at Ser^273^ is linked with the development of insulin resistance in mice; however, PPARγ protein degradation has not been associated with phosphorylation at these sites and cadmium did not promote phosphorylation of PPARγ at Ser^273^.

Selective ERK inhibitors have been used in clinical trials for the treatment of a variety of cancers. These compounds either disrupt ERK dimerization or bind to the active conformation site to inhibit kinase activity and phosphorylation of downstream kinases. BVD-523, the most advanced ERK inhibitor currently in phase I/II trial, showed ERK inhibition in whole blood from enrolled individuals and preliminary efficacy in patients with advanced solid tumors (38). ERK activation has been shown to be increased in mouse models of acute lung injury (52); however the mechanism by which its inhibition contributes to lung injury has not been determined. The data here indicate that ERK inhibition attenuates phosphorylation of PPARγ at Ser^112^ to maintain nuclear PPARγ expression and activation. Furthermore, administration of BVD-523 in cadmium-exposed or infected mice abrogated lung injury.

The thiazolidinedione class of PPARγ agonists, including rosiglitazone and pioglitazone, are used in the treatment of type 2 diabetes by improving insulin sensitivity. Administration of the PPARγ agonist rosiglitazone has been shown to promote the transition from the inflammatory phase to resolution and enhanced clearance of *Staphylococcus* *aureus* in mice (53). Although these agonists were beneficial in promoting insulin sensitization, these drugs are associated with many adverse effects, including weight gain, fluid retention, congestive heart failure, and bone fractures (54). This evidence indicates that alternative strategies for targeting PPARγ are warranted. The data presented here show that ERK regulates the phosphorylation of PPARγ at Ser^112^ to promote degradation of the protein. We have shown that targeting this posttranslational modification of PPARγ in MDMs may be a promising therapeutic target.

The induction of TNF-α in response to LPS has been shown to require ERK activation and the ERK pathway is essential for the transcriptional regulation of TNF-α (55). Our data support these observations. Cadmium-exposed individuals had increased levels of TNF-α in the BALF. This is especially interesting, in that these individuals also showed ERK activation in isolated lung macrophages. TNF-α plays an essential role in inflammation and immune homeostasis (56), but it has been implicated in many of the detrimental effects of chronic inflammation. Increased plasma levels of soluble TNF receptors are strongly associated with mortality and morbidity in patients with acute lung injury (57). Our data show that reduced lung injury is associated with reduced inflammation.

Our study has several limitations. Our single-cell RNA sequencing analysis was able to capture many cell types within the lung; however, we were not able to resolve TRAM and MDM subsets in exposed mice. Although interstitial macrophages showed limited expression of *Pparg* in exposed mice, we did not evaluate the contribution of these cells, as evidence suggests that monocytes give rise to interstitial macrophages and then differentiate into MDMs. Another limitation is that the composition of PM_2.5_ was not determined in this study. Because PM_2.5_ may contain various heavy metals, it is possible that other metals could share this mechanism. Additionally, as the cadmium-exposed participants recruited for this study had no recent or current evidence of infection, follow-up studies should assess their history of LRTIs. In aggregate, these observations suggest that the regulation of PPARγ in MDMs may be a novel target to protect against the severity of LRTIs secondary to lung injury mediated by air pollution.

## Methods

### Study participants.

We obtained human BAL cells from normal individuals and those from an area with high air cadmium levels. Normal volunteers had to meet the following criteria: (a) age between 18 and 75 years, (b) no history of cardiopulmonary disease or other chronic disease, (c) no prescription or nonprescription medication except oral contraceptives, (d) no recent or current evidence of infection, and (e) lifetime nonsmoker (<5 packs during lifetime). Cadmium-exposed participants had to meet the following criteria: (i) resident from the affected and control areas (zip codes 35207, 35217, and 35214) in North Birmingham, Alabama who have lived at these sites for a minimum of 2 years; (ii) FEV1 (forced expiratory volume in 1 second) greater than 1 liter; (iii) oxygen saturation greater than 90% at rest on room air; (iv) current nonsmoker; and (v) no recent or current evidence of infection. Fiberoptic bronchoscopy with BAL was performed after participants received local anesthesia. Three subsegments of the lung were lavaged (right middle lobe, right upper lobe, and lingual) with five 20-mL aliquots of normal saline, and the first aliquot in each was discarded. The percentage of macrophages was determined by Wright-Giemsa stain and varied from 90% to 98%.

### Mice.

WT C57BL/6J and *Ccr2^–/–^* mice were purchased from The Jackson Laboratory. *Pparg^fl/fl^* mice, a gift from Troy Randall, University of Alabama at Birmingham (UAB), were bred with mice containing Cre recombinase under the control of the lysozyme M promoter. The resulting *Pparg^–/–^* *Lyz2-cre* mice (referred to herein as *Pparg*^ΔM^) were generated by selective disruption of *Pparg* in the cells of the granulocyte/monocyte lineage as previously described (58). *Pparg^fl/fl^* mice were used as controls unless otherwise noted. Eight- to 12-week-old male and female mice were intratracheally (i.t.) administered 100 ng/kg of CdCl_2_ or saline, as a vehicle control, after being anesthetized with 3% isoflurane using a precision Fortec vaporizer. This dose was the determined cadmium concentration in BALF from mice exposed to cigarette smoke measured by inductively coupled plasma mass spectrometry (ICP-MS) (21). For in vivo bacterial infection studies, mice were administered sterile saline or 1 × 10^3^ *S*. *pneumoniae* (strain A66.1, type 3) i.t. and were euthanized after 15 days. Bacterial infections were performed 5 days after cadmium exposure. Mice were monitored every 4–6 hours after bacterial exposure for the duration of the exposure. BVD-523 (8.6 mg/kg; Selleck) was administered twice daily by oral gavage 1 day after S. *pneumoniae* infection until day 15. Dosing in mice was equivalent to the 600 mg dose used in a phase II clinical trial (ClinicalTrials.gov NCT04488003). Whole-lung lavage was performed and cytospins were generated to determine cell differential by Wright-Giemsa stain.

### Cell culture.

Human monocyte (THP-1) and mouse alveolar macrophage (MH-S) cell lines were obtained from American Type Culture Collection. Macrophages were maintained in RPMI 1640 media with 10% FBS and penicillin/streptomycin. All experiments were conducted in RPMI 1640 containing 0.5% FBS. Cells were treated with the MAPK/ERK kinase (MEK) inhibitor, U0126 (10 μM, 1 hour), prior to cadmium exposure. Cells were treated with vehicle or 50 μM CdCl_2_ for 3 hours or the indicated time, as previously described (27).

### Quantitative real-time PCR.

Total RNA was isolated, reverse transcribed, and quantitative real-time PCR was performed as described previously (58). Expression was calculated by the cycle threshold (ΔΔCT) method, normalized to β-actin, and expressed in arbitrary units. The following primer set was used: mouse *Arg1* 1: 5′-CAGAAGAATGGAAGAGTCAG-3′ and 5′-CAGATATGCAGGGAGTCACC-3′.

### Plasmids and transfection assays.

The pCMV-MEK1 and pCMV-HA-ERK2 (K/A) (ERK_DN_) plasmids (gifts from Roger Davis, University of Massachusetts Medical School, Worcester, Massachusetts, USA) have been previously described (33). The mouse PPARγ plasmid, a gift from Bruce Spiegelman, was purchased from Addgene (no. 8895) (32). Site-directed mutagenesis of PPARγ (S112A) was performed using the Q5 Site-Directed Mutagenesis Kit (E0552S, New England Biolabs). The correct reading frame and sequence were verified by the Heflin Center Genomics Core at UAB. Cells were transfected using X-treme GENE 9 Transfection Reagent (Roche Applied Scientific) according to the manufacturer’s protocol.

### Flow cytometry.

BAL cells were blocked with 1% BSA–containing TruStain fcX (anti–mouse CD16/CD32) antibody (101319, BioLegend), followed by staining with antibodies. Antibodies used were rat anti–mouse CD45-PE (12-0451-82, eBioscience), LIVE/DEAD–eFluor 506 (65-0866, Invitrogen), rat anti–mouse CD11b–APC-Cy7 (101225, BioLegend), anti–mouse CD64–PE-Cy7 (139313, BioLegend), rat anti–mouse Ly6G–Alexa Fluor 700 (561236, BD Biosciences), rat anti–mouse Siglec F–APC (155507, BioLegend), rat anti–mouse Ly6C–eFluor 450 (48-5932-82, Invitrogen), and rat anti–mouse MHC II–PerCP-Cy5.5 (562363, BD Biosciences). A hierarchical gating strategy was used to represent the TRAMs as CD45^+^CD11b^+/–^Ly6G^–^CD64^+^Ly6C^–^Siglec F^hi^ and MDMs as CD45^+^CD11b^+/–^Ly6G^–^CD64^+^Ly6C^–^Siglec F^lo^. Data were acquired on a FACSAria II or LSR II (BD Biosciences) using BD FACS DIVA software (version 8.0.1). Data were analyzed using FlowJo (FlowJo LLC) software (version 10.5.0).

### Single-cell RNA sequencing.

Single-cell suspensions were prepared as previously described (45) with slight modification. Lungs were perfused with 10 mL HBSS, removed, and infused with 1 mL digestion solution (Dispase with 0.2 mg/mL DNase I). Lungs were incubated at room temperature for 45 minutes with gentle agitation and tissue was gently teased apart with forceps into 1–2 mm fragments and incubated in digestion buffer for 15 minutes. The solution was filtered through a 70 μm cell strainer, washed with DMEM containing 5% FBS, centrifuged, and RBCs lysed with ACK lysing buffer. Resulting single-cell suspensions were filtered twice through a 40 μm cell strainer with DMEM containing 5% FBS. Cells were counted after staining with 7-AAD for exclusion of nonviable cells; cell viability exceeded 95%.

The 10× Genomics CellRanger software (version 7.0.0), mkfastq, was used to create the fastq files from the sequencer. After fastq file generation, CellRanger counts were used to align the raw sequence reads to the mouse reference genome (mm10). The matrix table created using the counts was then loaded into the R package Seurat (version 4.1.1), which allows for selection and filtration of cells based on quality control metrics, data normalization and scaling, and detection of highly variable genes (59). The Seurat vignette (https://satijalab.org/seurat/articles/pbmc3k_tutorial.html) was followed to create the Seurat data matrix object. In brief, we retained all genes expressed in more than 3 cells and cells with at least 200 detected genes. Cells with mitochondrial gene percentages greater than 10% and unique gene counts greater than 6000 or less than 200 were discarded. The data were normalized using Seurat’s NormalizeData function, which uses a global-scaling normalization method, LogNormalize, to normalize the gene expression measurements for each cell to the total gene expression. The result was multiplied by a scale factor of 1 × 10^4^ and the result was log-transformed. Highly variable genes were then identified using the function FindVariableFeatures in Seurat. SelectIntegrationFeatures was used to select features that were repeatedly variable across the samples for integration. Anchors were identified using FindIntegrationAnchors, which uses the above integration features. These anchors were then used to integrate the samples together with IntegrateData. Variation arising from library size and percentage of mitochondrial genes was regressed using the Seurat function ScaleData. PCA was performed on the variable genes as input and significant principal components determined on the basis of the Seurat JackStraw function. The first 30 principal components were selected as input for uniform manifold approximation and projection (UMAP) using FindNeighbors, FindClusters (resolution = 0.8), and RunUMAP in Seurat. To help aid in identifying cell types, SingleR (version 1.10.0) was used to annotate the identified clusters and manually verified with FindConservedMarkers (46). To identify differentially expressed genes in each cell cluster, we used Seurat’s FindMarkers function on normalized gene expression.

### Confocal imaging.

Macrophages and BAL cells were fixed with 4% paraformaldehyde in PBS for 45 minutes at room temperature, followed by permeabilization for 3 minutes and incubation in PBS containing 5% BSA for 45 minutes. Cells were incubated with anti–rabbit PPARγ (A0270, Abclonal), anti–rabbit p-PPARγ (04-816-I, Sigma-Aldrich), or anti–mouse p-ERK (675502, BioLegend) and goat anti–rabbit IgG-FITC (4030-02, Southern Biotech) or goat anti–mouse IgG, human ads-TRITC (1030-03, Southern Biotech) and counterstained with DAPI. A Nikon A1 confocal microscope was utilized for imaging.

### ICP-MS.

Cd levels in BAL samples were measured using ICP-MS (Agilent 7500a). Aliquots (500 μL) were prepared as previously described (21). Samples were analyzed in sextuplicate, and the concentration was calculated using a standard calibration curve. All dilution solutions used for analysis were treated with Chelex 100 resin to remove cations.

### Lung bacterial burden.

Bacterial burden was determined in excised lungs as previously described (21). Lungs were homogenized in PBS and serial, 3-fold dilutions were made and plated on blood agar plates containing 4 μg/mL gentamicin sulfate. The CFUs were determined approximately 16 hours after plating and incubation; results are expressed as CFU per mL of lung tissue.

### Isolation of nuclei.

Nuclear isolation was performed by resuspending cells in a lysis buffer (10 mM HEPES, 10 mM KCl, 2 mM MgCl_2_, and 2 mM EDTA) for 15 minutes on ice. Nonidet P-40 (10%) was added to lyse the cells, and the cells were centrifuged at 4°C at 21,000*g*. The nuclear pellet was resuspended in an extraction buffer (50 mM HEPES, 50 mM KCl, 300 mM NaCl, 0.1 mM EDTA, and 10% glycerol) for 20 minutes on ice. After centrifuging at 4°C at 21,000*g*, the supernatant was collected as nuclear extract (33).

### Immunoblot analysis.

Primary antibodies used were anti–Lamin A/C (catalog 2032), rabbit monoclonal anti–p-p38 MAPK (Thr180/Tyr182) (catalog 9215), anti–p-SAPK/JNK (Thr183/Tyr185) (catalog 9255) (all Cell Signaling Technology); anti–β-actin (catalog A5441) and anti–p-PPARγ S112 (catalog 04-816-I) (both Sigma-Aldrich); anti–p-ERK (sc-7383, Santa Cruz Biotechnology); anti-PPARγ (A0270, Abclonal); and anti-PPARα (catalog PAa-822A), anti-PPARδ (PA1-823A), and anti–p-PPARγ S273 (catalog BS-4888R) (all Thermo Fisher Scientific). See complete unedited blots in the supplemental material.

### Immunoprecipitation.

Immunoprecipitation of PPARγ was performed by lysing 10 million cells in buffer supplemented with EDTA-free protease inhibitor cocktail. Beads from a Dynabeads Protein G Kit (10007D, Invitrogen) were incubated with anti-PPARγ antibody (16643-1-AP, ProteinTech) to form bead-antibody complexes. To avoid co-elution of the bound antibody, the complex was crosslinked with disuccinimidyl suberate. Equal amounts of total protein from supernatant were incubated with the bead-antibody complexes overnight at 4°C. The complexes were washed 3 times and His-tagged proteins eluted. Purification of His-tagged proteins was performed as previously described (21).

### ELISA.

IL-6, IL-8, and TNF-α expression was determined in BALF using ELISA kits (R&D Systems) according to the manufacturer’s instructions.

### Albumin.

Albumin levels were determined in BALF using the human (Millipore) or mouse Albumin ELISA Kit (Immunology Consultants Laboratory) according to the manufacturer’s protocol. Samples were diluted 1:500,000.

### Materials.

U0126 and MG-132 were purchased from Sigma Chemical Company.

### Statistics.

Statistical comparisons were performed using a Student’s *t* test when only 2 groups of data are presented, or 1-way ANOVA with Tukey’s post hoc test or 2-way ANOVA followed by Bonferroni’s post hoc test when multiple data groups are present. All data are expressed as mean ± SEM and a *P* value of less than 0.05 was considered significant. GraphPad Prism statistical software was used for all analysis.

### Data availability.

Data have been submitted to the NCBI Gene Expression Omnibus database (GEO) with GEO accession number GSE225386.

### Study approval.

We obtained BAL cells under approved protocols (300004607 and 300001124) by the Human Subjects Institutional Review Board of the UAB. Human BAL specimens were used for research only. All participants provided prior written consent to participate in the study. Animal experiments were approved by the UAB Institutional Animal Care and Use Committee under protocols 21969 and 21149 and were performed in accordance with the NIH *Guide for the Care and Use of Laboratory Animals* (National Academies Press, 2011).

## Author contributions

JLLC and ABC developed the concept and design of the study. JLLC, SL, and JMP assisted with conducting experiments. JLLC, SL, JMP, SEL, DKC, KS, VBA, and ABC acquired data. JLLC, SEL, VBA, and ABC provided reagents. JLLC, JMP, SEL, DKC, MLG, and ABC provided analysis and interpretation of experiments and results. JLLC and ABC wrote the manuscript.

## Supplementary Material

Supplemental data

## Version 1 03/16/2023

In-Press Preview

## Version 2 05/08/2023

Electronic publication
