# Supplementary material for: Impaired PPARγ activation by cadmium exacerbates infection-induced lung injury
Source: JCI Insight. 2023 May 8;8(9):e166608. doi: 10.1172/jci.insight.166608 (PMC10243824; doi:10.1172/jci.insight.166608)

**Supplemental Information**

# Impaired PPARɣ activation by cadmium exacerbates infection-induced lung injury

Jennifer L. Larson-Casey, Shanrun Liu, Jennifer M. Pyles, Suzanne E. Lapi, Komal Saleem, Veena B. Antony, Manuel Lora Gonzalez, David K. Crossman, and A. Brent Carter

**Supplemental Figures and Legends:**

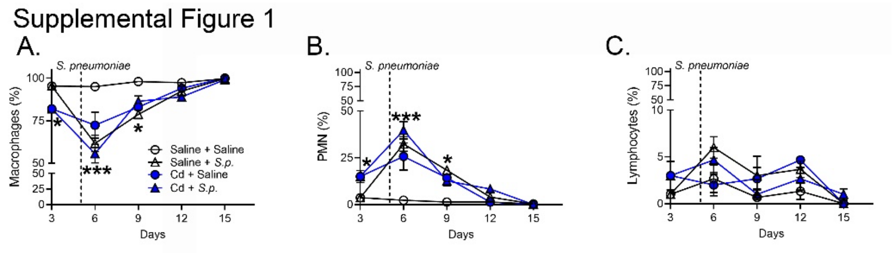


**Supplemental Figure 1. Cell differentials from exposed mice.** Cell differential to identify (**A**) macrophages, (**B**) neutrophils (PMN), and (**C**) lymphocytes. $n = 3$. *, $p < 0.05$; ***, $p < 0.0001$ vs. Saline + Saline. Values shown as mean ± S.E.M. Two-way AVOVA, Tukey's post hoc.

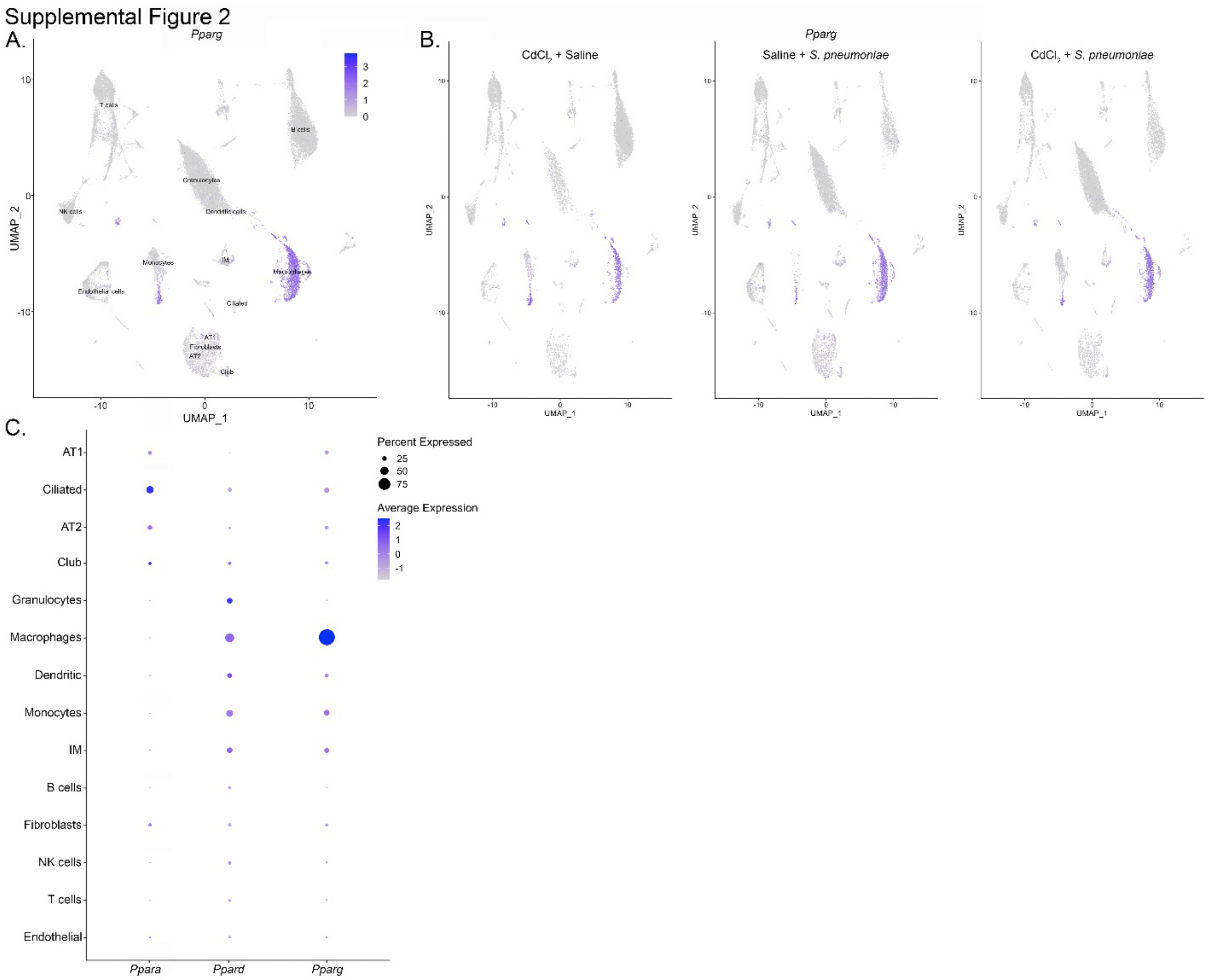


**Supplemental Figure 2. Cell clusters expressing *Pparg* from exposed mice.** *Pparg* expression on UMAP plot of (**A**) all cells and (**B**) after individual exposure. (**C**) Dot plots of *Ppara, Ppard,* and *Pparg* expression in all cell clusters.

Supplemental Figure 3

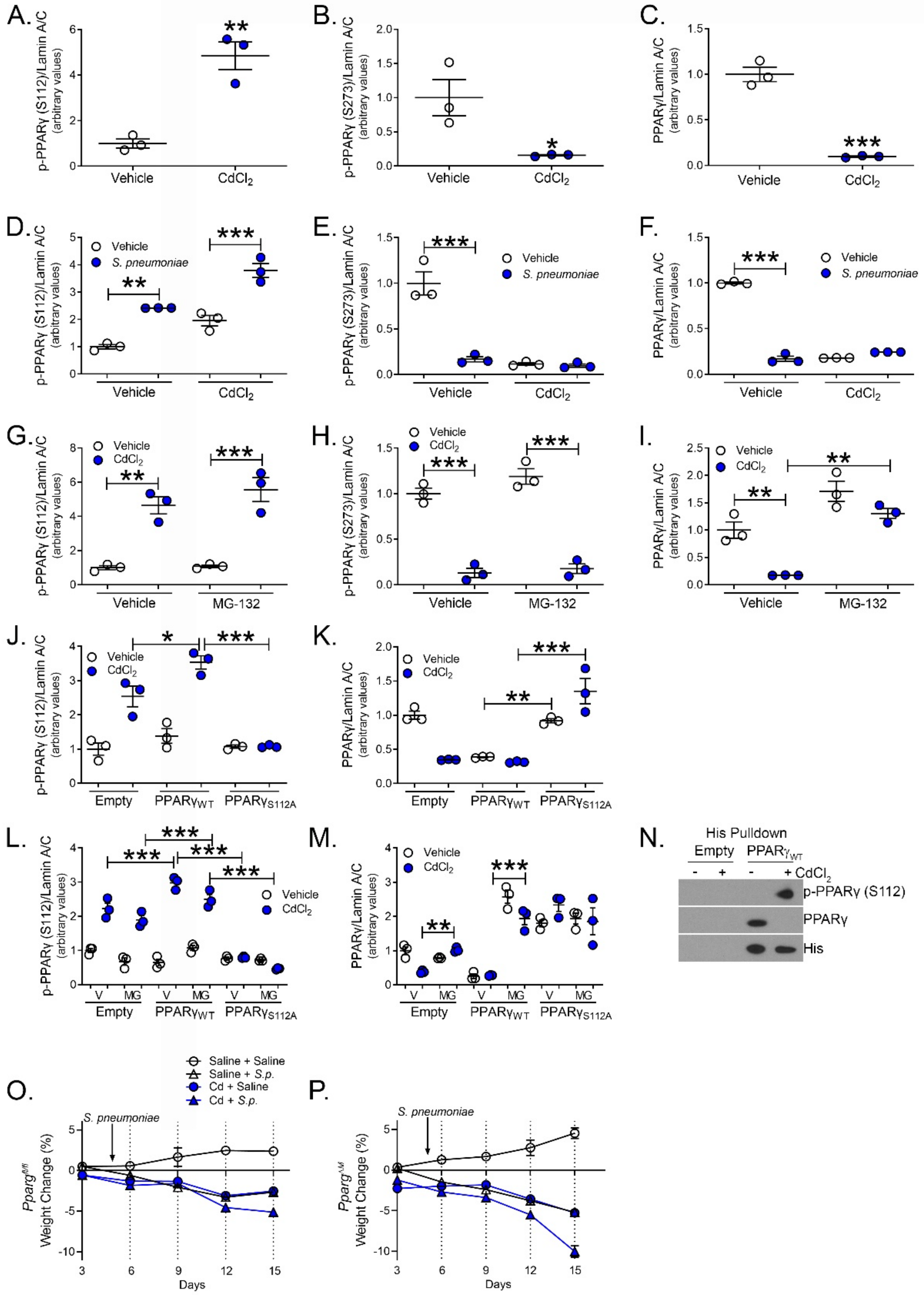


**Supplemental Figure 3. Cadmium regulates PPARɣ phosphorylation at Ser[112] resulting in greater lung injury.** Statistical quantification of (**A**) p-PPARɣ (S112), (**B**) p-PPARɣ (S273), and (**C**) PPARɣ from the nuclear immunoblot analysis of THP-1 cells exposed to $CdCl_2$ (50 µM, 3h)

$n = 3$. Statistical quantification of (**D**) p-PPARɣ (S112), (**E**) p-PPARɣ (S273), and (**F**) PPARɣ from the nuclear immunoblot analysis from BAL cells from exposed WT mice $n = 3$. Statistical quantification of (**G**) p-PPARɣ (S112), (**H**) p-PPARɣ (S273), and (**I**) PPARɣ from immunoblot analysis in isolated nuclear extract of THP-1 cells treated with vehicle or MG-132 (20 µM, 6h) and saline or $CdCl_2$ (50 µM, 3h) $n = 3$. Statistical quantification of (**J**) p-PPARɣ (S112) and (**K**) PPARɣ from the nuclear immunoblot analysis of THP-1 cells expressing empty, $PPARɣ_{WT}$, or $PPARɣ_{S112A}$ treated with saline or $CdCl_2$ $n = 3$. Statistical quantification of (**L**) p-PPARɣ (S112) and (**M**) PPARɣ from the nuclear immunoblot analysis of THP-1 cells expressing empty, $PPARɣ_{WT}$, or $PPARɣ_{S112A}$ treated with saline or $CdCl_2$ and vehicle or MG-132. $n = 3$. (**N**) THP-1 ells transfected with empty or $PPARɣ_{WT}$-His and treated with saline or $CdCl_2$ (50 µM, 3h). PPARɣ-His was purified by pull down and immunoprecipitation for PPARɣ. Percent weight change of exposed (**O**) $Pparg^{fl/fl}$ and (**P**) $Pparg^{\Delta M}$ mice. $n = 3\text{-}5$. *, $p < 0.05$; **, $p < 0.001$; ***, $p < 0.0001$. Values shown as mean ± S.E.M. Student's $t$-test used in A-C. One-way AVOVA, Tukey's post hoc.

Supplemental Figure 4

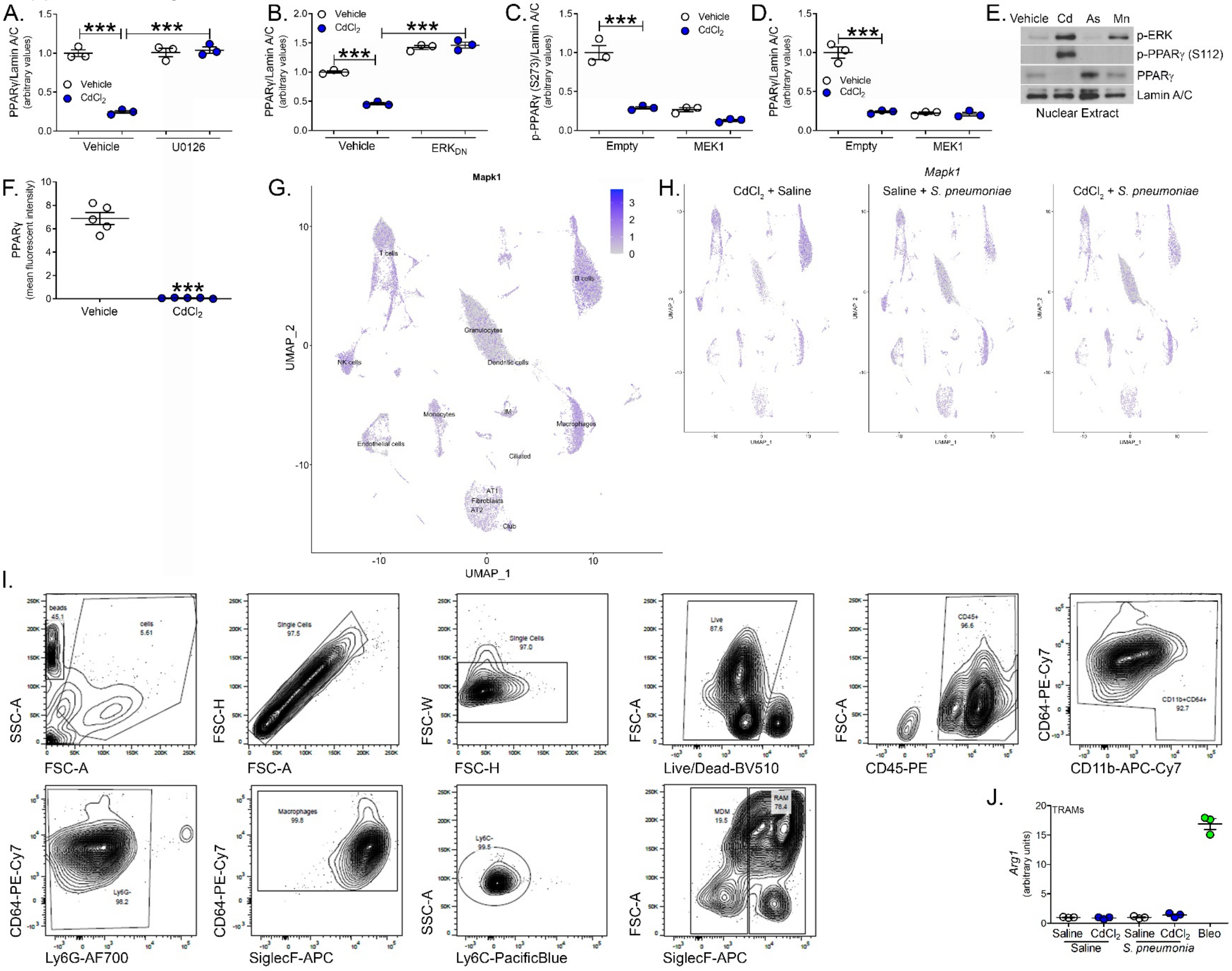

**Supplemental Figure 4. Cell clusters expressing *Mapk1* from exposed mice.** (**A**) Statistical quantification of PPARɣ from the nuclear immunoblot analysis of THP-1 cells exposed to vehicle or U0126 (10 μM, 1h) and $CdCl_2$ (50 μM, 3h) $n = 3$. (**B**) Statistical quantification of PPARɣ from the nuclear immunoblot analysis of THP-1 cells transfected with empty or $ERK_{DN}$ and exposed to saline or $CdCl_2$ $n = 3$. Statistical quantification of (**C**) p-PPARɣ (S273) and (**D**) PPARɣ from the nuclear immunoblot analysis of THP-1 cells transfected with empty or MEK1 and exposed to saline or $CdCl_2$ $n = 3$. (**E**) Immunoblot analysis of THP-1 cells exposed to $CdCl_2$ (50 μM, 3h), $NaAsO_3$ (100 μM, 3h), or $MnCl_2$ (200 μM, 3h). (**F**) Statistical quantification of representative confocal imaging of exposed MH-S cells for PPARɣ staining $n = 5$. *Mapk1* expression on UMAP plot of (**G**) all cells and (**H**) after individual exposure. (**I**) Representative flow gating strategy from isolated BAL after cadmium exposure. Tissue resident alveolar macrophages (TRAM, $CD45^{+}CD11b^{+/-}Ly6G^{-}CD64^{+}Ly6c^{-}Siglec\ F^{hi}$) and monocyte-derived macrophages (MDM, $CD45^{+}CD11b^{+/-}Ly6G^{-}CD64^{+}Ly6c^{-}Siglec\ F^{low}$). (**J**) Arginine 1 mRNA expression in TRAMs from exposed WT mice, bleomycin (bleo) BAL cells used as positive control. $n = 3$. ***, $p < 0.0001$. Values shown as mean ± S.E.M. Student's *t*-test used in F. One-way AVOVA, Tukey's post hoc.

Supplemental Figure 5

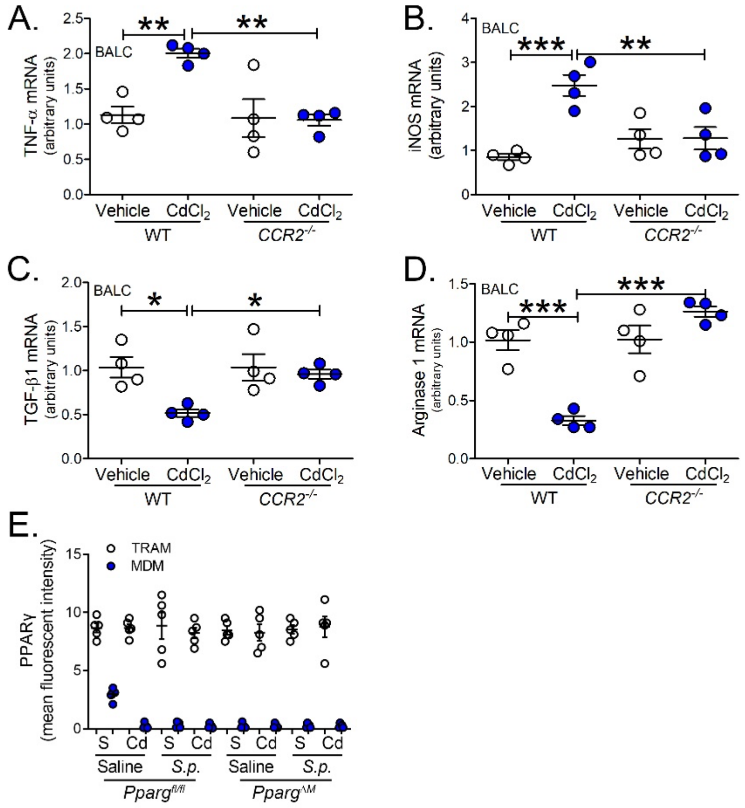


**Supplemental Figure 5. *CCR2$^{-/-}$* mice show inhibition of cadmium-mediated inflammation.** WT and *CCR2$^{-/-}$* mice were exposed to saline or $CdCl_2$ (100ng/kg) by i.t. administration. mRNA expression in isolated BAL cells of (**A**) TNF-α, (**B**) iNOS, (**C**) TGF-β1, and (**D**) Arg1. $n = 4$. Statistical quantification of confocal imaging from (**E**) PPARɣ staining $n = 5$. *, $p < 0.05$; **, $p < 0.001$; ***, $p < 0.0001$. Values shown as mean ± S.E.M. One-way AVOVA, Tukey's post hoc.

Supplemental Figure 6

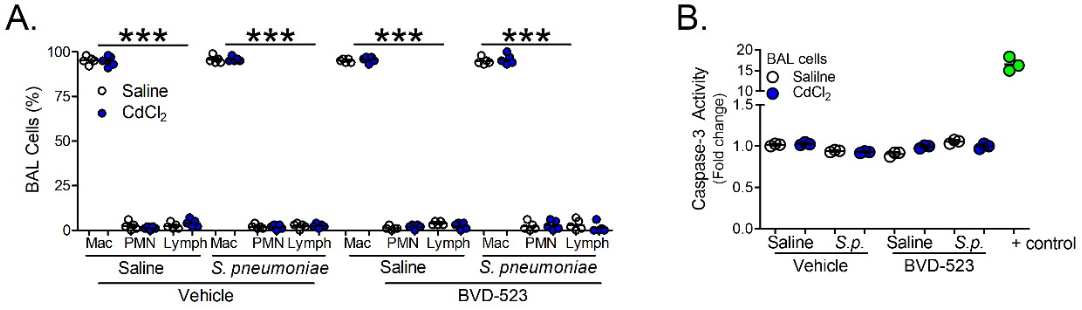


**Supplemental Figure 6. ERK inhibition does not alter cell type present in BAL fluid.** (**A**) Cell differential to identify macrophages (mac), neutrophils (PMN), and lymphocytes (lymph). $n = 5$. (**B**) Caspase-3 activity in BAL cells from exposed mice. $n = 3$. Positive control = 5 µM Rhodamine 110 reference standard. ***, $p < 0.0001$. Values shown as mean ± S.E.M. One-way AVOVA, Tukey’s post hoc.

Figure 3

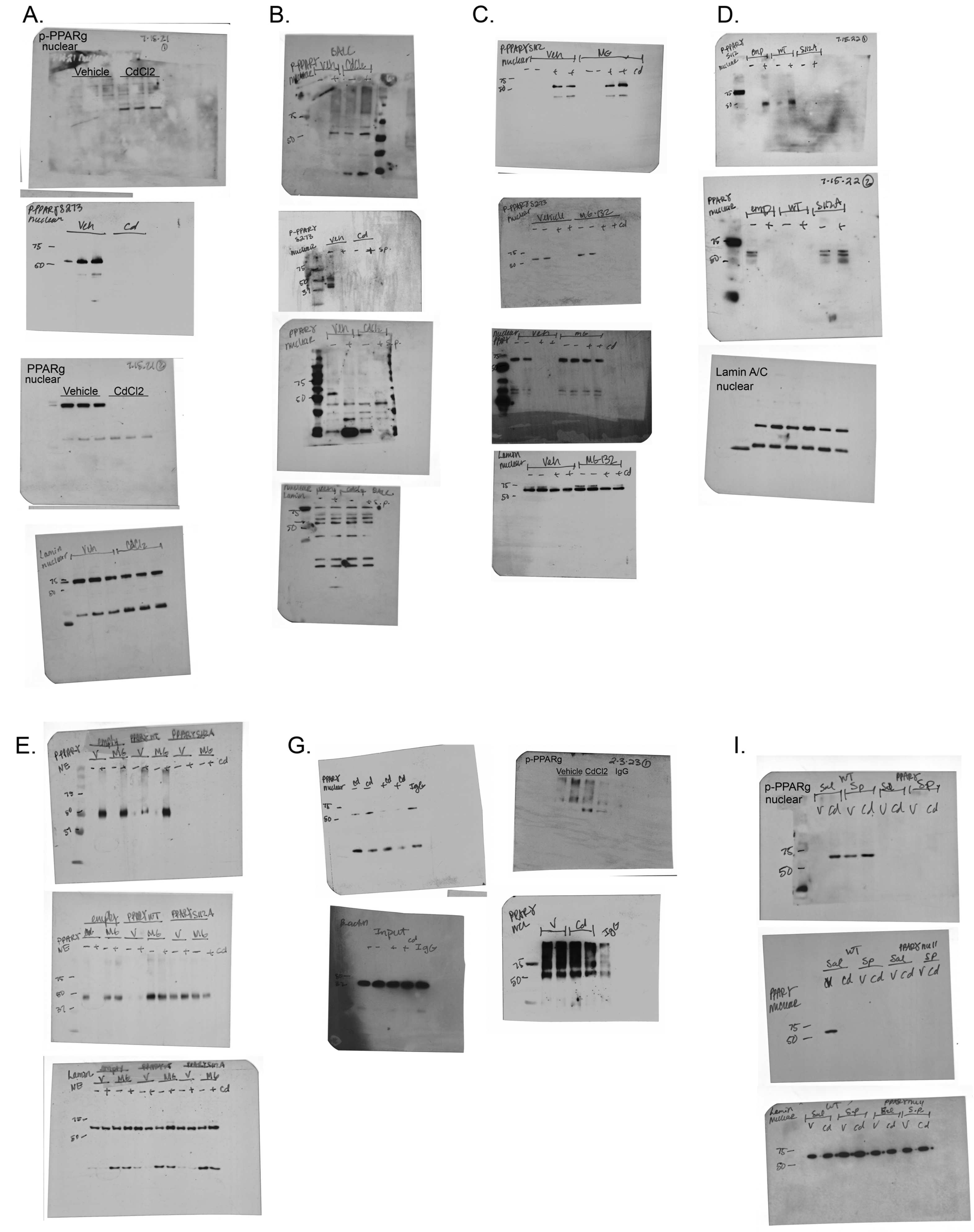
A.
B.
C.
D.
E.
G.
I.
p-PPARg nuclear
Vehicle CdCl2
PPARg nuclear
Vehicle CdCl2
Lamin A/C nuclear
p-PPARg
Vehicle CdCl2 IgG
p-PPARg nuclear

Figure 4

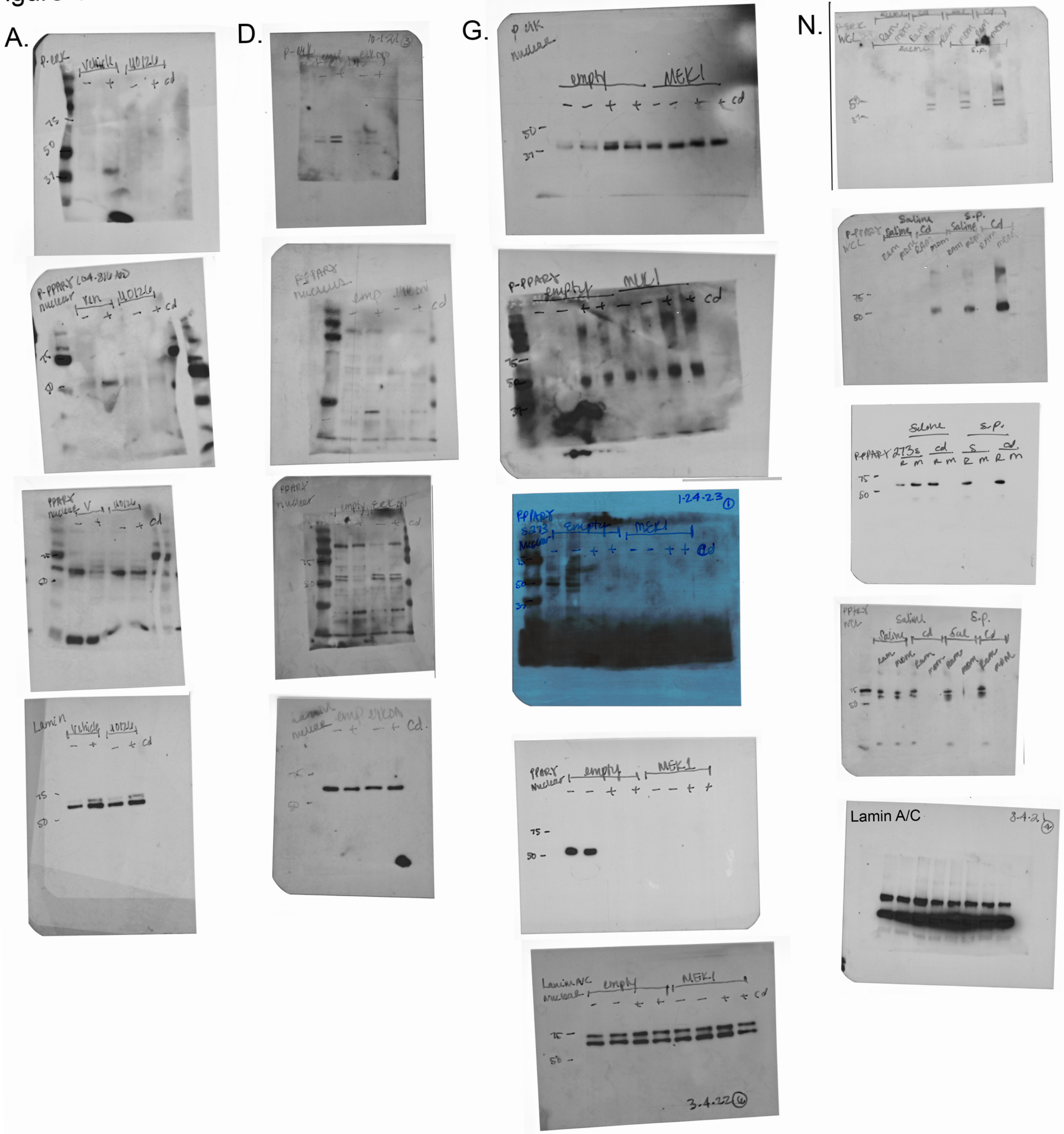

Figure 6

C.

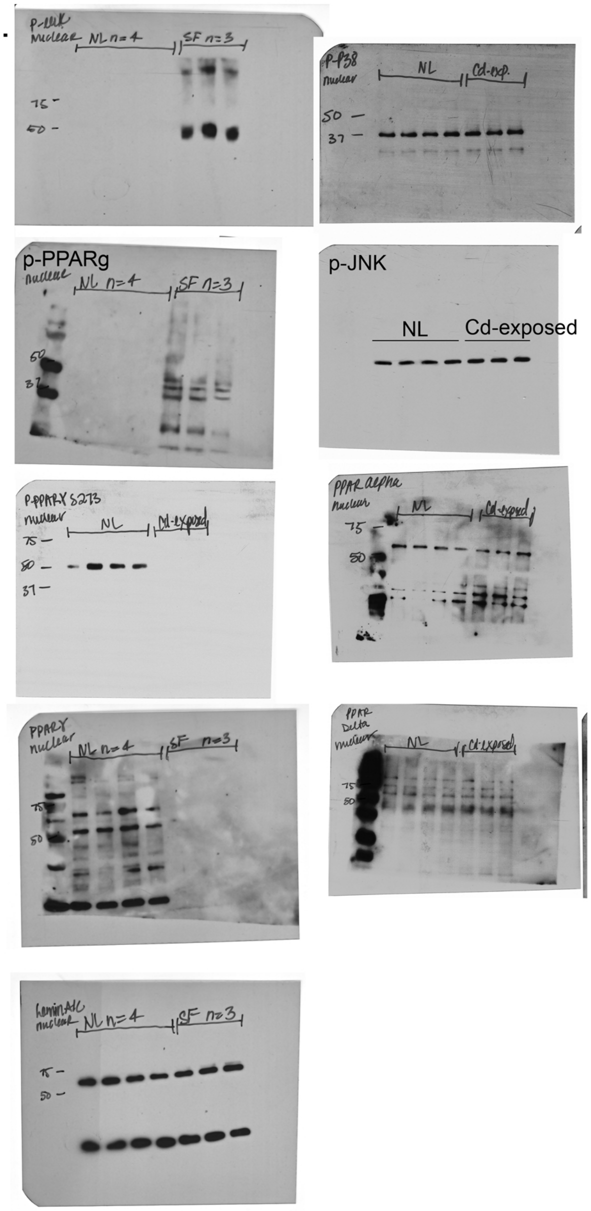


Figure 7

A.

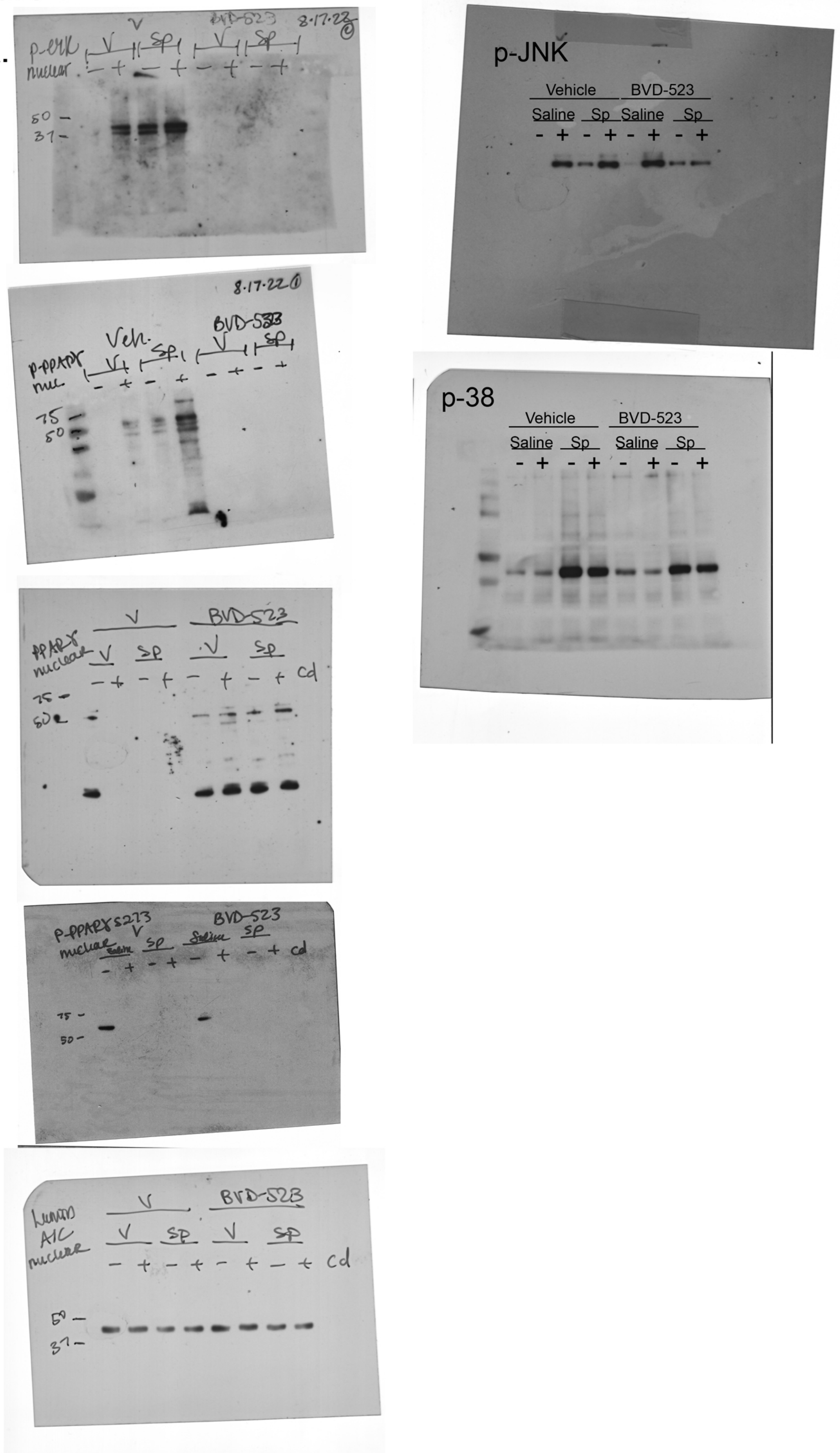

## Supplemental Figure 3

N.

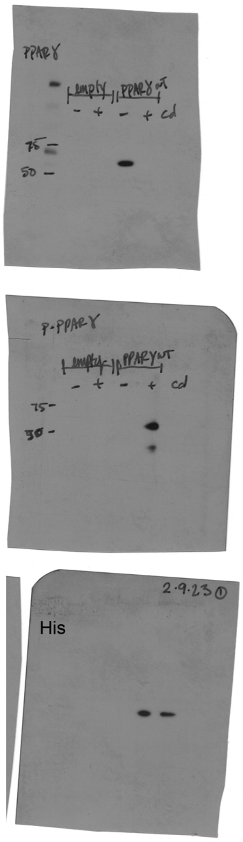


## Supplemental Figure 4

E.

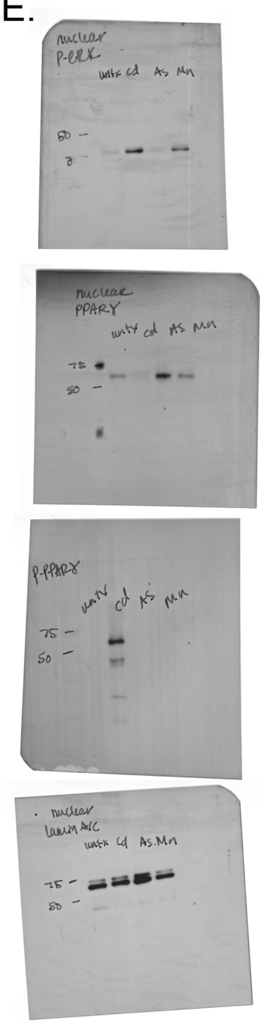

Supplement: Supplemental data [file jciinsight-8-166608-s008.pdf]
